# Novel approach to enhance *Bradyrhizobium diazoefficiens* nodulation through continuous induction of ROS by manganese ferrite nanomaterials in soybean

**DOI:** 10.1186/s12951-022-01372-2

**Published:** 2022-03-31

**Authors:** Jun Ma, Yi Zhou, Jiaying Li, Zhiyong Song, Heyou Han

**Affiliations:** 1 State Key Laboratory of Agricultural Microbiology, College of Life Science and Technology, Huazhong Agricultural University, No. 1 Shizishan Street, Hongshan District, Wuhan, 430070 Hubei China; 2 State Key Laboratory of Agricultural Microbiology, College of Science, Huazhong Agricultural University, No. 1 Shizishan Street, Hongshan District, Wuhan, 430070 Hubei China

**Keywords:** Reactive oxygen species, Nodulation, Autoregulation of nodulation, Manganese ferrite, Soybean

## Abstract

**Background:**

The study of symbiotic nitrogen fixation between (SNF) legumes and rhizobia has always been a hot frontier in scientific research. Nanotechnology provides a new strategy for biological nitrogen fixation research. However, how to construct abiotic nano-structure-biological system, using the special properties of nanomaterials, to realize the self-enhancement of biological nitrogen fixation capacity is important.

**Results:**

In order to construct a more efficient SNF system, in this study, we applied manganese ferrite nanoparticles (MF-NPs) with sustainable diatomic catalysis to produce reactive oxygen species (ROS), thus regulating the nodulation pathway and increasing the number of nodules in soybean (*Glycine max*), eventually enhancing symbiotic nitrogen fixation. Symbiosis cultivation of MF-NPs and soybean plants resulted in 50.85% and 61.4% increase in nodule weight and number, respectively, thus inducing a 151.36% nitrogen fixation efficiency increase, finally leading to a 25.70% biomass accumulation increase despite no substantial effect on the nitrogenase activity per unit. Transcriptome sequencing analysis showed that of 36 differentially expressed genes (DEGs), 31 DEGs related to soybean nodulation were upregulated in late rhizobium inoculation stage (12 d), indicating that the increase of nodules was derived from nodule-related genes (Nod-R) continuous inductions by MF-NPs.

**Conclusions:**

Our results indicated that the nodule number could be effectively increased by extending the nodulation period without threatening the vegetative growth of plants or triggering the autoregulation of nodulation (AON) pathway. This study provides an effective strategy for induction of super-conventional nodulation.

**Graphical Abstract:**

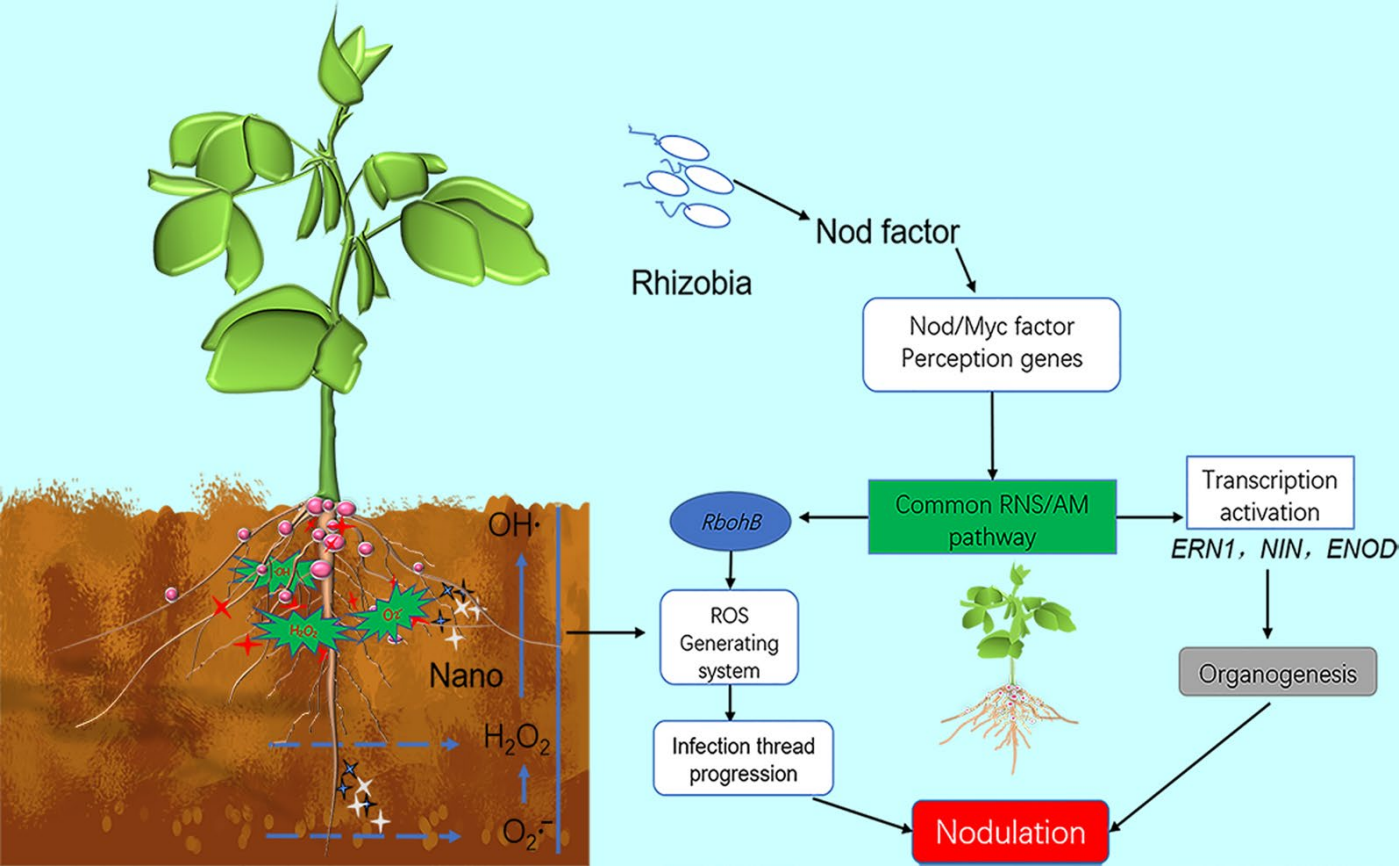

**Supplementary Information:**

The online version contains supplementary material available at 10.1186/s12951-022-01372-2.

## Background

Due to the long-term evolution of nitrogen-fixing organisms in the biosphere, the biological nitrogen fixation (BNF) is considered as one of the most environmentally friendly and sustainable methods to obtain nitrogen [1]. As the main source of human plant protein, soybean plays an indispensable role in human food supply [2]. By promoting soybean nodulation and enhancing total nitrogen fixation is considered as one of the most effective and convenient methods to increase yield. In recent years, the application of nanotechnology in agriculture has gradually attracted attention [3], and nanotechnology may be a potential breakthrough for sustainable agricultural development [4, 5]. Increasing the nodulation of legumes through functional nanoproducts is a breakthrough attempt.

Reactive oxygen species **(**ROS), as highly reactive oxygen derivatives, includes superoxide anion (O_2_^∙−^), hydroxyl radicals(∙OH), hydroperoxyl radicals (∙HO_2_), and hydrogen peroxides (H_2_O_2_). These radicals are produced during aerobic metabolism under biotic and abiotic stresses [6, 7]. Despite its toxicity, ROS can serve as signals to trigger metabolic regulations in response to environment stresses. ROS is produced during nodulation in legumes under the induction of nodulation factors (NFs) and participates in infection formation, contributing to cell wall reconstruction, matrix glycoprotein (MGP) cross-linking, and actin microfilament nucleation and branching [8]. ROS regulates nodulation in conjunction with Ca^2+^ through spatial and temporal alterations [9]. Symbiotic production of ROS is primarily accomplished through cytoplasmic membrane NADPH oxidases respiratory burst oxidase homologue (*Rbohs*) [10, 11], and *Rbohs* overexpression stimulates rhizobium infection and nodule formation, resulting in an increase in the number of symbiotic microsomes and nitrogen fixation efficiency [12]. Conversely, the inhibition of *Rbohs* decreases ROS production and downregulates the expression of rhizobium-induced peroxidases (*RIPs*), *NIN*, and *ENOD2* [13, 14]. Collectively, ROS in legumes is a group of signaling molecules regulating nodulation. In the process of nodulation, in order to control the stress caused by excessive nodules on the plant, a microRNA (miRNA), as a feedback regulation switch, can reduce the amount of *GmNNC1* protein by shearing *GmNNC1* mRNA, and alleviate the inhibitory effect of transcription repressor *GmNNC1* on *GmENOD40,* thus allowing *GmEOND40* to further activate the signal transduction pathway, finally initiating the development of nodules [15]. When there are too many root nodules, root-derived nodulation-specific CLAVATA/ESR-related (CLE) peptides *GmRIC1* and *GmRIC2* [16] induce the autoregulation of nodulation (AON) through shoot-derived inhibitor (SDI) such as cytokinin to inhibit the expression of *miR172c*, thereby avoiding excessive nodulation of soybeans [15, 17].

The reaction between H_2_O_2_ and Fe^2+^ is called Fenton reaction (FTR). The factors affecting FTR include pH, temperature, reaction time, H_2_O_2_ concentration, and Fe^2+^ concentration, The FTR reaction equation was as follows (Eq. 1):

1 $${\text{Fe}}^{{{2} + }} + {\text{ H}}_{{2}} {\text{O}}_{{2}} \to {\text{Fe}}^{{{3} + }} + \, \left( {{\text{OH}}} \right) - \, + {\text{OH}\cdot}$$

Further research has revealed that some materials such as, manganese-based, cobalt-based, and copper-based materials show high efficiency in FTR-like. Nanomaterials-mediated FTR is also widely used in fields such as biomedicine [18, 19] and environmental protection [20–24]. The capacity to produce ∙OH in FTR-like between Mn^2+^ and H_2_O_2_ is lower than that in the Fenton reaction between H_2_O_2_ and Fe^2+^, but the former (with Mn^2+^ participation) can extend induction time of ROS, and adding a certain amount of Mn^2+^ to the FTR system can significantly promote FTR efficienc. It has been reported that the reaction of Mn^2+^ and H_2_O_2_ can produce O_2_^∙−^ [25]. Fe^3+^ produced in FTR can be reduced to Fe^2+^ to form a sustainable FTR under the mediation of O_2_^∙−^. The corresponding reaction equations were as follows [26, 27] (2–4)

2 $${\text{H}}_{{2}} {\text{O}}_{{2}} + {\text{Mn}}^{{{2} + }} /{\text{Fe}}^{{{2} + }} \to {\text{Mn}}^{{{3} + }} /{\text{Fe}}^{{{3} + }} + {\text{OH}} - + {\cdot\text{OH}}$$

3 $${\cdot\text{OH}} + {\text{ H}}_{{2}} {\text{O}}_{{2}} \to {\text{HOO}\cdot} + {\text{H}}_{{2}} {\text{O}}$$

4 $${\text{HOO}\cdot} \to {\text{H}}^{ + } + {\text{O}}_{{2}}^{ \cdot - }$$

In the iron-manganese diatomic FTR system, the scavenging capacity of ∙OH and O_2_^∙−^ is inhibited since the Mn^3+^ generated in the system is reduced by the Fe^2+^ ion. Previous studies have revealed that the reduction of Mn^3+^ by Fe^2+^ is thermodynamically favorable [28, 29]. The corresponding reaction Eqs. (5–7) were as follows.

5 $${\text{Fe}}^{{{3} + }} + {\text{ 1e }} - {\text{ Fe}}^{{{2} + }} ,{\text{ E}}^{ \circ } = \, 0.{\text{77 V}}$$

6 $${\text{Mn}}^{{{3} + }} + {\text{ 1e }} - {\text{ Mn}}^{{{2} + }} ,{\text{ E}}^{ \circ } = { 1}.{\text{51 V}}$$

7 $${\text{Fe}}^{{{2} + }} + {\text{Mn}}^{{{3} + }} - {\text{ Fe}}^{{{3} + }} + {\text{Mn}}^{{{2} + }} ,{\text{ E}}^{ \circ } = \, 0.{\text{73 V}}$$

The higher reduction capacity of Mn^3+^ than Fe^3+^ promotes the reduction of Mn^3+^and the production of Mn^2+^, thus extending the catalytic reaction time of H_2_O_2_ decomposition. Therefore, the FTR mediated by the MF-NP diatomic system is recyclable and sustainable.

All above evidence supports that nanoparticles can induce ROS production through FTR. Based on this, we assumed that legume nodulation could be induced by ROS generated from FTR with optimized conditions to extend nodulation time, thus achieving the same desirable effect as regulating *Rbohs* gene expression. The sustainability of nanoparticle-induced FTR generating ROS and extension of the nodulation period overcome the adverse impact of excessive nodulation on plant vegetative growth. Our findings provide a reference for developing strategies of excessive nodulation.

## Materials and methods

### Substrate preparation

MnFe_2_O_4_ (MF-NPs, Purity: 99.5%) and MnO_2_ (M-NPs, Purity: 99.9%) were purchased from Shanghai Bike New Material Technology Co., Ltd.; Fe_2_O_3_ (F-NPs, Purity: 98.0%) from Aladdin Reagent Co., Ltd. (Shanghai, China); FeCl_2_ and MnCl_2_ (analytical purity) from Sinopharm Chemical Reagent Co., Ltd. (Shanghai, China). This experiment was performed with the soybean variety “*Williams 82*” and rhizobium *USDA110* strain. Before *USDA110* inoculation, nanomaterials (MF-NPs, M-NPs, F-NPs, and F-NPs+M-NPs) were dissolved in 1.2 L ultrapure water under ultrasonication at the concentrations of 0.1, 1, 10, 50, and 100 mg L^−1^ with the metal ion concentration in ionic materials (FeCl_2_ at 1.1, 10.10, 54.98, 109.95 mg L^−1^and MnCl_2_ at 0.55, 5.45, 27.27, 54.55 mg L^−1^) consistent with that in MF-NPs. After mixing materials and 6L vermiculite and stirring thoroughly, the mixture was divided into groups according to concentrations, sterilized at 121 °C for 20 min, and transferred to an ultra violet (UV)-sterilized plug tray after complete cooling. The control group (CK) was not mixed with any material in ultrapure water.

### Planting conditions

The soybean seeds were surface-sterilized in sodium hypochlorite under stirring for 4 min, rinsed 6 times with sterile water, transferred into a blank agar petri dish, and incubated at 25 °C in a dark incubator for germination. The germinated soybeans were planted into sterile plug tray containing matrixes with a planting depth of 3 cm. After planting, each plug tray with 32 plants in total was watered with 2 L of nitrogen-free nutrient solution every three days. Next, the seedlings were cultured at a greenhouse (14 h-light/10 h-dark cycle at a light intensity of 150 μmol m^−2^ s^−2^).

### Rhizobium inoculation

After activation of rhizobia, a single colony was collected and inoculated into YMA liquid medium for 72 h at 28 °C in a shaker (150 rpm) for further use. Afterwards, rhizobia were washed with sterile water. The absorbance value (OD_600_) of the bacterial liquid was adjusted 0.5. After 1-week planting of the seedlings, the base matrix around each plant root was inoculated with 1 mL bacterial liquid.

### Sampling time

On day 4 post inoculation, root samples were collected to observe the curling of root hairs and the staining of H_2_O_2_ and O_2_^∙−^. The roots were sampled on day 4, 8, and 12 post rhizobium inoculation for transcriptome sequencing analysis. On day 12, 16, and 20, the samples were collected to observe the nodule development status. On day 28, samples were collected to evaluate the growth indexes including nodule weight, nodule number, plant height, and root length. After the nodule was fully mature (on day 28), the root system with nodules was collected to determine the nitrogenase activity, and the mature nodule sections were observed to evaluate the development morphology of the symbiosis zone and the biological activity of rhizobia.

### Nitrogenase activity assay

The nitrogenase activity was measured as our previously study described [30]. The difference is that the sample used for GC detection in this study was 200 μL. The standard curve data (Additional file 1: Fig. S24) were converted to C_2_H_4_ concentration, C_2_H_4_ yield (μmol min^−1^) was normalized to mass (μmol h^−1^ g^−1^), and nitrogenase activity was calculated by the following equation:

$$\partial = \frac{M}{m \times t}$$

where ∂ indicates nitrogenase activity (μmol h^−1^ g^−1^); *M,* ethylene production (μmol); *m,* nodule weight (g); *t,* reaction time (h).

### H_2_O_2_ decomposition assay

A total of 2.5 mM H_2_O_2_ was mixed separately with MF-NPs, F-NPs, M-NPs, F-NPs+M-NPs, MnCl_2_, or FeCl_2_ in 20 mL PBS at room temperature. Then, 1 mL reaction solution was centrifuged at 10,000r for 3 min to collect the supernatant for test using the H_2_O_2_ content assay kit (Solarbio, Beijing, China). After the reaction was completed, the absorbance of the solution at 415 nm was measured to evaluate the H_2_O_2_ concentration. The above operation was repeated at 10 min, 30 min, 1 h, 3 h, 5 h, 12 h, 24 h, 3 d, 7 d, and 14 d during the reaction.

### Hydroxyl radical generation

The nanomaterials (at 1.25 mg mL^−1^ concentration) and ionic materials (with the ion concentration in ionic materials consistent with that of MF-NPs) were separately prepared into mother liquid, and 1 M methylene blue (MB) solution was prepared. Subsequently, 100 μL material mother liquid (nanomaterials or ionic materials) and 200 μL of MB solution were mixed with 2 mL of 0.3% H_2_O_2_ solution under shaking. After reaction for 0, 6, 12, 18, 24, 30, 36, 42, and 48 h, spectral scanning was performed, and OD_660_ was measured for quantitative analysis.

### Detection of H_2_O_2_ and O_2_∙^−^

H_2_O_2_ and O_2_^∙−^ in plant roots in situ were visualized by DAB and NBT staining, as previously described [31]. Briefly, the root H_2_O_2_ content was analyzed by staining the roots with 0.1 mg mL^−1^ DAB in 50 mM Tris buffer (pH 5.0). The O_2_^∙−^ content was measured after root incubation with 2 mM NBT in 20 mM K-phosphate containing 0.1 M NaCl (pH 6.1). Root images were taken under brightfield light.

### Transcriptomic analyses of soybean

On day 4, 8, and 12 post *Rhizobium* inoculation into vermiculite media with or without 10 mg L^−1^ MF-NPs (as CK), the whole root tissues were collected from the plants, immediately put into an Eppendorf tube, and stored in liquid nitrogen. Total RNA was extracted using an plant RNA extraction kit (TransGen Biotech, Beijing, China). The obtained total RNA was sequenced on Illumina NovaSeq sequencing platform, and the mRNA sequencing libraries were constructed (MetWare Biological Science and Technology Co., Ltd. Wuhan, China). Subsequently, the sequencing reads were mapped to the *Glycine max* genome using hisat2 software. The differentially expressed genes (DEGs) were identified using DESeq software with *P* value < 0.05, the false discovery rate (FDR) < 0.05, and a |log2 Fold Change| of ≥ 1 as thresholds.

### Bacterial live and dead stain

Frozen microtome (Leica CM1950) was used for microsection to observe the live and dead bacteria in nodules. Each slice was stained with the mixture of SYTO_9_ and propidium iodide (PI) stains and held in the dark for 30 min, followed by observation under a fluorescence microscope. The bacteria with intact cell membranes showed green fluorescence, in contrast to red fluorescence for bacteria with damaged membranes.

### Statistical analysis

All the data were presented as the mean ± standard deviation (± SD). The experiments were performed in triplicates. Significant differences between groups were determined by independent sample *t-test* and statistical differences were analyzed by applying the one-way ANOVA. The significance levels were determined at *P < 0.05, **P < 0.01, and ***P < 0.001.

### KEGG pathway analysis of DEGs

KEGG pathway analysis method is explained by Irudayaraj [32].

## Results

### Characterization of nanoparticles

**Fig. 1:**
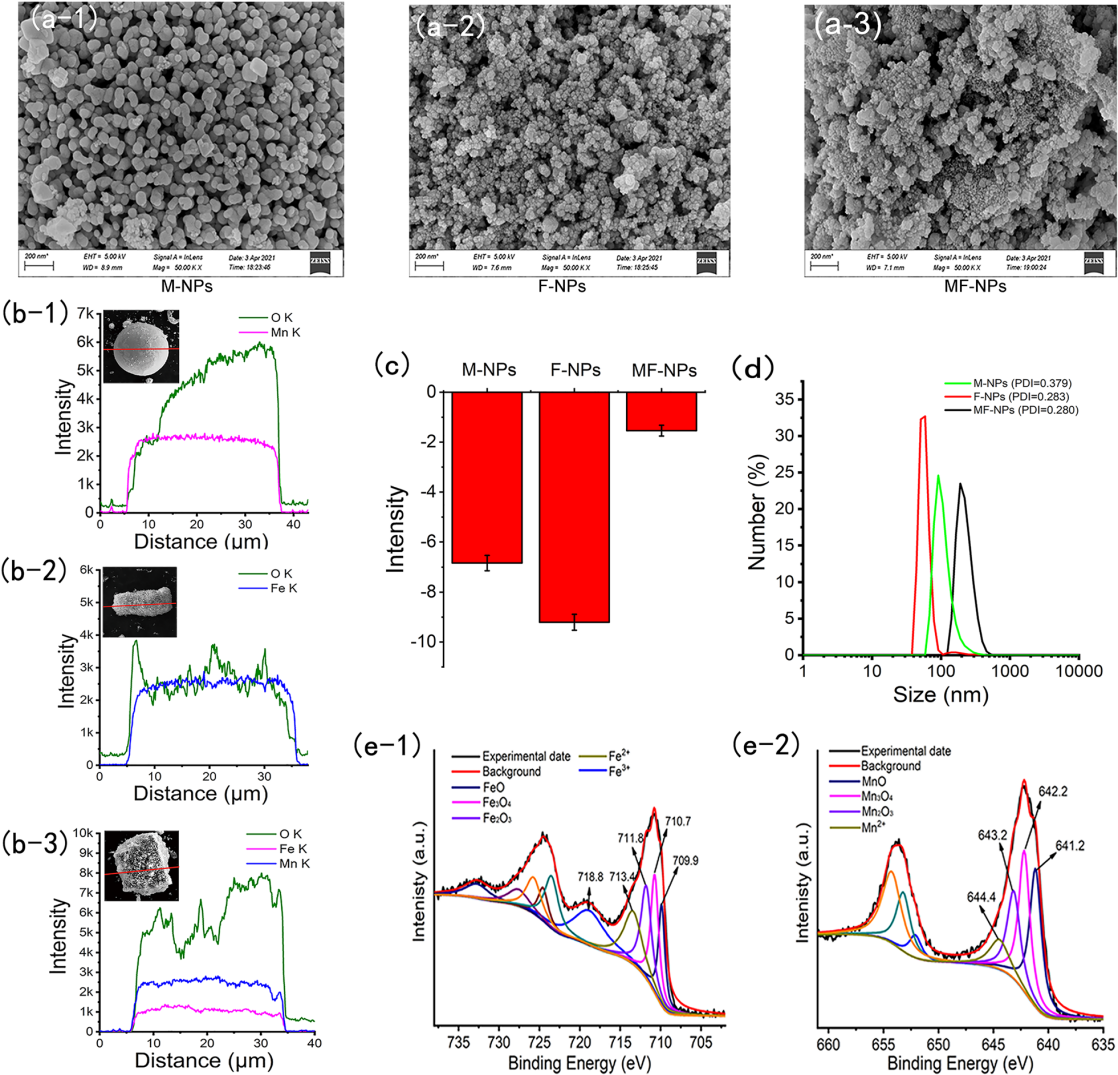
Material characterization. **a** SEM. **b** Element line scan. **c** Zeta-potential (n = 3). **d** Size distribution. **e** XPS spectra of MF-NPs in Fe2p region (**e-1**) and Mn2p region (**e-2**)

In this study, nanomaterials (MnO_2_ nanoparticles, M-NPs; Fe_2_O_3_ nanoparticles, F-NPs; and MnFe_2_O_4_ nanoparticles, MF-NPs) and conventional ionic materials (FeCl_2_ and MnCl_2_) were derived from ordinary commercial production. Nanomaterials were characterized accordingly. Scanning electron microscopy (SEM) (Fig. 1a) and transmission electron microscopy (TEM) (Additional file 1: Fig. S1) results indicated that nanomaterials used in this study were irregular granules. Line scanning results indicated that the content of manganese and oxygen in M-NPs was 40.12% and 59.88%, respectively (Fig. 1b), that the content of iron and oxygen in F-NPs was 52.14% and 47.86%, respectively, and that the content of iron, manganese, and oxygen in MF-NPs was 29.64%, 10.84%, and 59.52% (Additional file 1: Table S1). The zeta potential value (Fig. 1c) of M-NPs, F-NPs, and MF-NPs in deionized water (pH = 6.5) was − 6.8 ± 0.3 mV, − 9.2 ± 0.3 mV, and − 1.5 ± 0.2 mV, respectively, and the average particle size distribution was 223.43 nm for MF-NPs, 59.34 nm for F-NPs, and 108.71 for M-NPs (Fig. 1d). Fourier transform infrared (FTIR) spectroscopy showed no specific surface functional groups in MF-NPs (Additional file 1: Fig. S2). The photoelectron spectrum of MF-NPs showed that Fe 2p3/2 peaks for FeO, Fe_3_O_4,_ and Fe_2_O_3_ appeared at binding energies of 709.9 eV, 710.7 eV, and 711.8 eV, respectively [33, 34], coupled with the satellite peaks at binding energies of 713.4 eV and 718.8 eV in the Fe 2p region (Fig. 1e). The Mn 2p3/2 peaks for MnO, Mn_3_O_4_, and Mn_2_O_3_ appeared at binding energies of 641.2 eV, 642.2 eV, and 643.2 eV, respectively, coupled with satellite peaks at a binding energy of 644.4 eV [33, 35].

### Synergistic ROS generation of MF-NPs

**Fig. 2:**
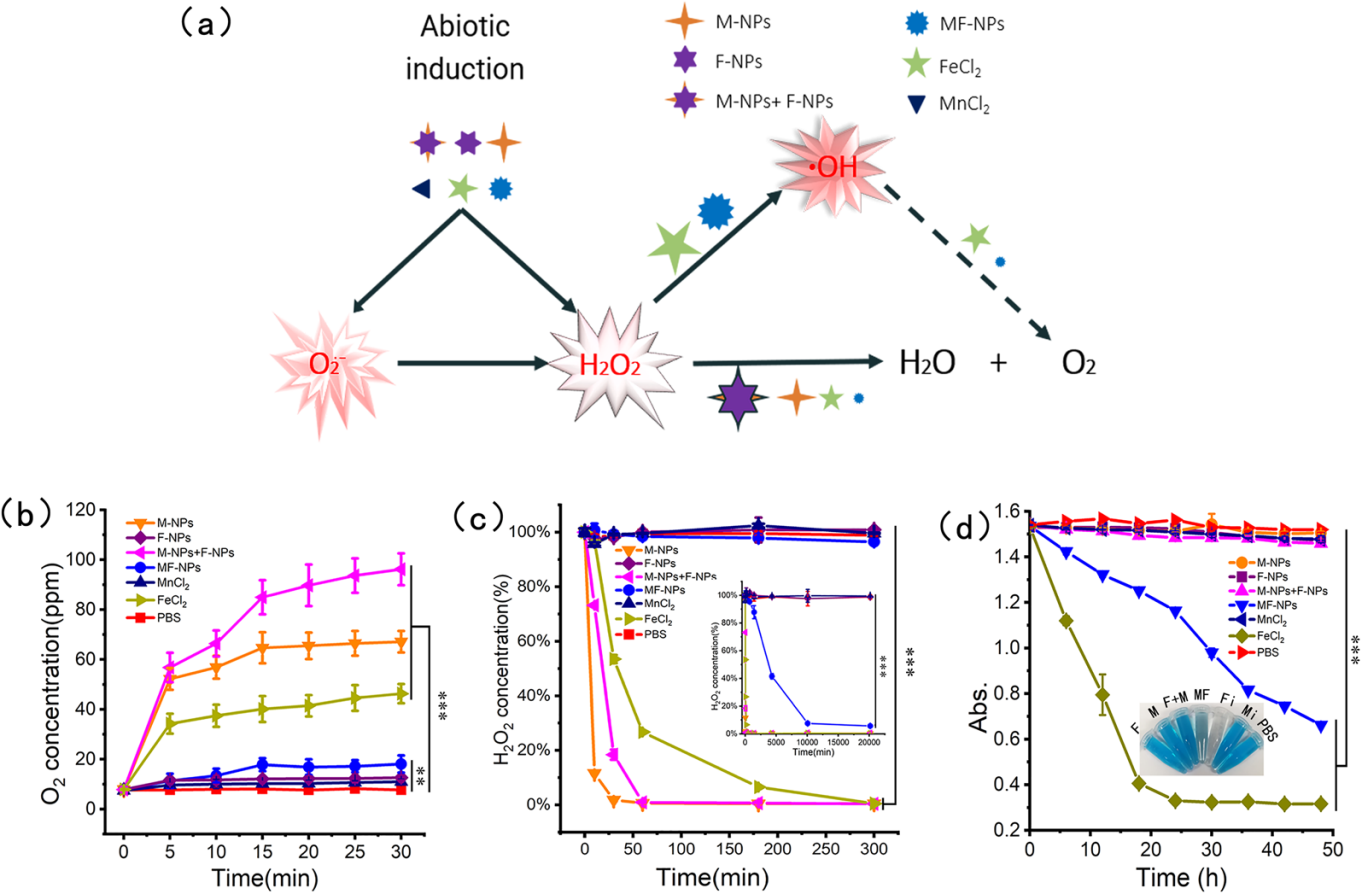
H_2_O_2_ scavenging and ·OH generation by iron-based and manganese-based MF-NPs. **a** Mechanism of ROS generation by NPs. **b** Curves of O_2_ generation in H_2_O_2_ solution in the presence of NPs and conventional materials (CMs) at pH 6.5(n = 3). **c** Curves of H_2_O_2_ degradation in the presence of MF-NPs and related materials at pH 6.5 (n = 3). **d** Time-dependent hydroxyl radical generation in the presence of NPs and CMs containing H_2_O_2_ at pH 6.5 (n = 3) with the fading degree of MB as detection indicator

The mechanism by which MF-NPs catalyzed ROS production was studied by comparing the Mn-based nano-oxides, Fe-based nano-oxides, and ionic compounds (Fig. 2a). The determination results of the increase value in dissolved oxygen and the number of O_2_ bubbles showed that under M-NPs, M-NPs+F-NPs, and FeCl_2_ exposures, O_2_ generation capability was increased within 30 min, which was faster than under MF-NP exposure (Fig. 2b and Additional file 1: Fig. S3). A time-dependent H_2_O_2_ assay at pH 6.5 indicated that nano-sized M-NPs and M-NPs+F-NPs decomposed almost all H_2_O_2_ within 1 h, and FeCl_2_ also achieved similar effects within 5 h (Fig. 2c). Under MF-NP exposure, about 10% H_2_O_2_ residue was still detected 1 week later. Only a weak H_2_O_2_ decomposition was observed under F-NP and FeCl_2_ exposures. After adding H_2_O_2_ to reaction system (pH 6.5), ∙OH concentration in the presence of NPs was evaluated by detecting the fading degree of methylene blue (MB) induced by ∙OH (Fig. 2d). The ∙OH yield under MF-NP exposure was significantly higher than that under other exposures except FeCl_2_, for FeCl_2_, Fe^2+^ which could be rapidly converted to Fe^3+^, but could not be sustainable. Similar results were observed in the spectral curve of the time dynamic response (Additional file 1: Fig. S4). The results showed that M-NPs, F-NPs+M-NPs, and FeCl_2_ could convert intermediate ∙OH to generate more O_2_, whereas MF-NPs only a few convert. Compared with FeCl_2_, MF-NPs had a lasting hydroxyl radical production efficiency in FTR system containing Fe^2+^ and Mn^2+^.

### Response to MF-NPs exposure and Fenton reaction

**Fig. 3:**
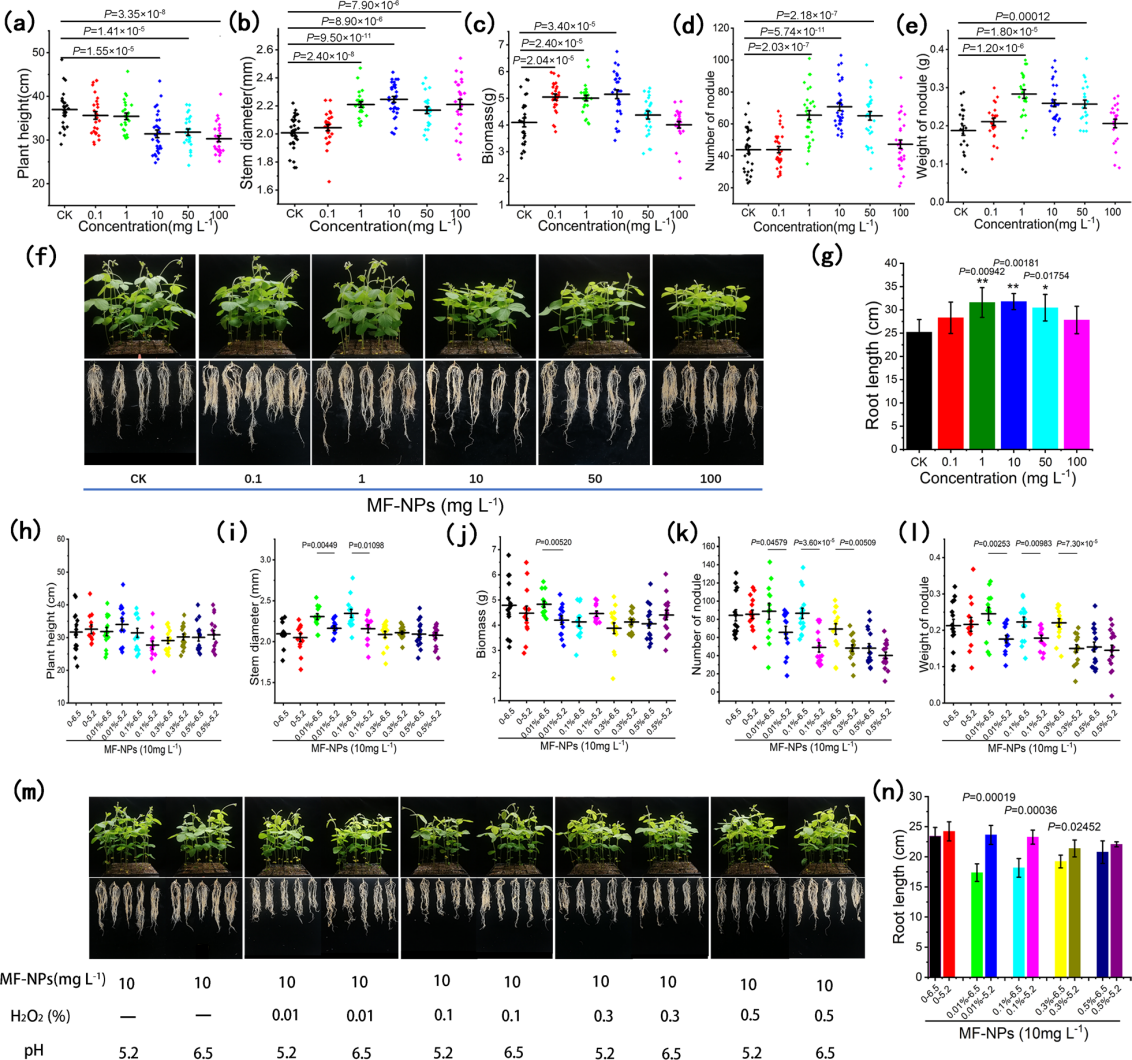
Plant response to different concentration MF-NPs exposure and Fenton reaction. **a**–**e** Responses to various concentrations of MF-NPs exposure: **a** Plant height (n ≥ 25). **b** Stem diameter (n ≥ 23). **c** Total biomass (n ≥ 24). **d** Nodule number (n ≥ 31). **e** Nodule weight(n ≥ 22). **f**, **g** Plants exposed to 0, 0.1, 1, 10, 50, or 100 mg L^−1^ of MF-NPs for 4 weeks. **f** Root length phenotype (experiment number, n = 5). **g** Root length under various concentrations of MF-NPs exposure. **h**–**l** Plant growth performance under10 mg L^−1^MF-NPs exposure in response to Fenton reaction induced by H_2_O_2_ at pH 6.5 and 5.2. **h** Plant height (n ≥ 12). **i** Stem diameter (n ≥ 13). **j** Total biomass (n ≥ 13). **k** Nodule number (n ≥ 13). **l** Nodule weight (n ≥ 13). **m** Growth phenotype(n = 5). **n** Root length (n = 5)

To evaluate the response of soybean growth to different nanomaterials, soybeans were planted in substrates containing different nanomaterials and their physiological and nitrogen fixation indexes were analyzed at different inoculation time points. The plant height analysis indicated a general decrease in the height of the soybean plants treated with a certain concentration of manganese-based materials (M-NPs+F-NPs, M-NPs, MF-NPs, MnCl_2_), especially that treated with MF-NPs. However, almost no obvious change in plant height was observed for the treatment with F-NPs and FeCl_2_ at all concentrations (Additional file 1: Figs. S5, S6). The stem diameter (Additional file 1: Fig. S7) analysis showed a completely opposite trend to that of plant height, with a larger diameter in the groups treated with M-NPs+F-NPs, M-NPs, MF-NPs, and MnCl_2_ than in the groups treated with F-NPs and FeCl_2_, and the most significant stem diameter difference was observed in the MF-NP treatment group at all concentrations, which might be due to dwarfing induced by ROS. Root length analysis indicated that root growth was obviously promoted by all the treatments except F-NP treatment at certain concentrations, which might be due to the advantages of iron and manganese as massive element in plant physiological growth (Additional file 1: Figs. S8, S9). Biomass analysis (Additional file 1: Fig. S10) showed only a significant increase in MF-NP and MnCl_2_ treatment groups, which was consistent with the increasing trend of nodule number (Additional file 1: Fig. S11) and weight (Additional file 1: Fig. S12), suggesting that biomass increase might be related to the increase in nitrogen nutrition. Under the treatment with different concentrations of MF-NPs, the plant exhibited a height decrease of 15.04%, 13.99%, and 18.10% in response to 10 mg L^−1^, 50 mg L^−1^, and 100 mg L^−1^ MF-NPs, respectively (Fig. 3a); a stem diameter increase of 10.4% at 1 mg L^−1^, 11.88% at 10 mg L^−1^, 8.05% at 50 mg L^−1^, and 10.06% at 100 mg L^−1^) (Fig. 3b); a biomass increase at 0.1 mg L^−1^, 1 mg L^−1^, and 10 mg L^−1^, with a maximum increase of 25.70% (1.05 g) at 10 mg L^−1^ (Fig. 3c); a maximum nodule weight increase of 0.28 g at 1 mg L^−1^ (Fig. 3e) and a maximum nodule number increase of 71 at 10 mg L^−1^ with various degrees of increase at 1 mg L^−1^, 10 mg L^−1^, and 50 mg L^−1^ (Fig. 3d). The growth phenotype investigation showed that the root length exhibited a significant increase of 25.36%, 26.19%, and 20.92% at 1 mg L^−1^, 10 mg L^−1^ and 50 mg L^−1^, respectively, and that plant growth promotion effect was weakened at a high concentration of 50 or 100 mg L^−1^(Fig. 3f, g).

The response of soybean growth and nitrogen fixation indicators to Fenton reaction was examined at the optimal promoting concentration (10 mg L^−1^) under two different acidity matrixes (pH 6.5) and (pH 5.2) conditions. As shown in Fig. 3m, in the case of low pH-induced ROS redundancy, no significant difference was observed in plant height (Fig. 3h). Under 0.01% and 0.1% H_2_O_2_ exposure, the stem diameter at pH 6.5 matrix was respectively 6.62% and 8.71% higher than that at pH 5.2, and under 0.01% and 0.1% H_2_O_2_ exposure at pH 6.5, the stem diameter was 10.05% and 11.89% higher than that without H_2_O_2_ exposure at the same pH value (Fig. 3i). Biomass exhibited an increase of 15.06% under 0.01% H_2_O_2_ exposure at pH 6.5 matrix relative to pH 5.2 (Fig. 3j). Meanwhile, the nodule number (Fig. 3k) and weight (Fig. 3l) under 0.01%, 0.1%, and 0.3% H_2_O_2_ exposure at pH 6.5 were 1.35, 1.78, 1.43 (number) and 1.40, 1.25, and 1.47 (weight) times as much as those at pH 5.2, respectively. The weight under 0.01% H_2_O_2_ exposure at pH 6.5 was 15.32% higher than that without H_2_O_2_ exposure at pH 6.5. Root length (Fig. 3m, n) showed significant difference between pH 5.2 and pH 6.5 at low H_2_O_2_ concentrations (0.01% and 0.1%) and tended to be stable with the increase of H_2_O_2_ concentration, but excessively high H_2_O_2_ concentration (> 0.1%) induced the suppression of the root length (independent of pH) and all growth indicators (regardless strong or weak acid).

### Seedling roots response to MF-NP exposure

**Fig. 4:**
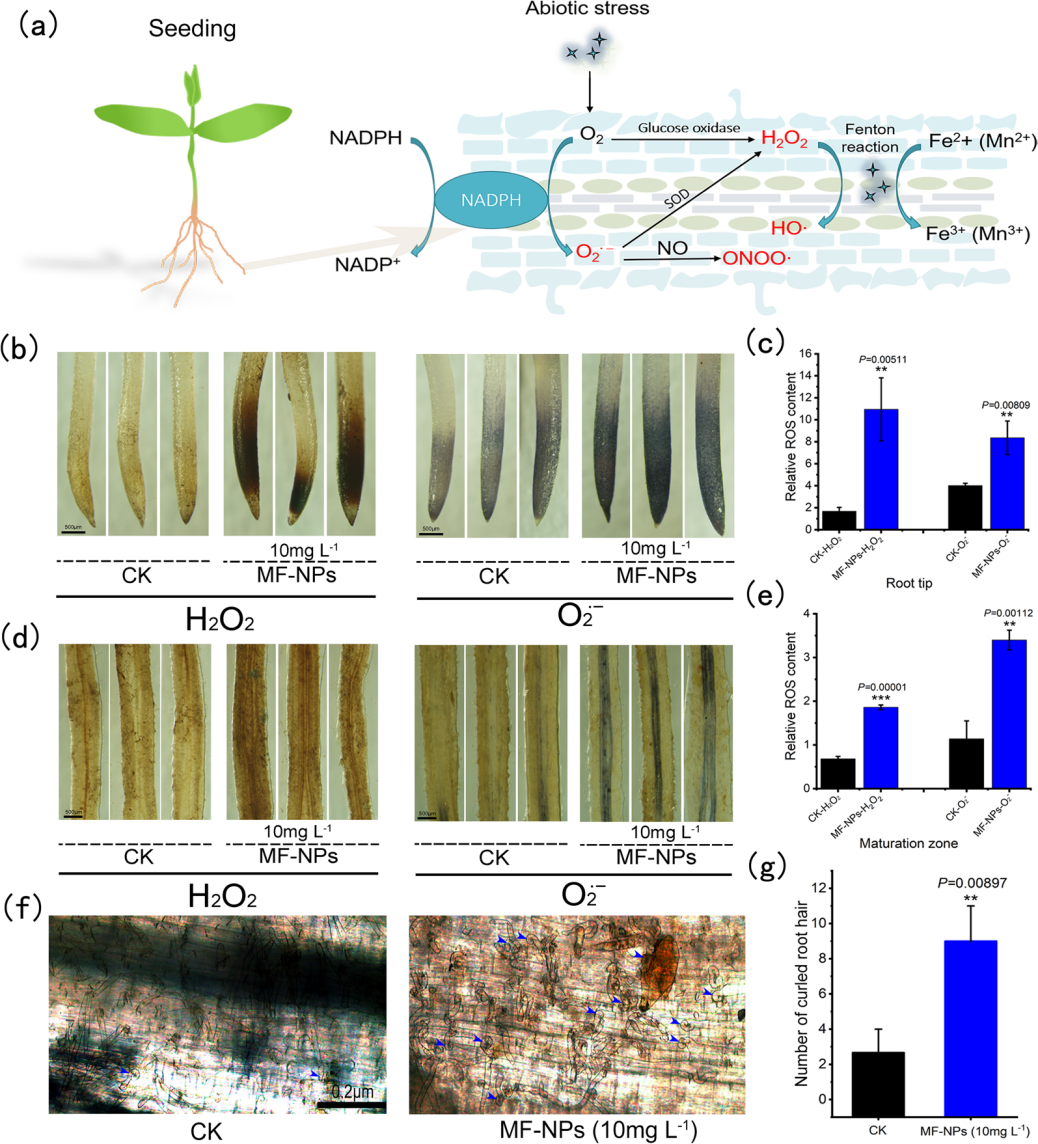
ROS distribution in roots and root hair curling. **a** ROS production mediated by MF-NPs under abiotic stress. **b**, **c** Root tip phenotype and relative ROS content in root tip on day 7 post inoculation. root tip (n = 3) and (n = 3) grown in vermiculite (10 mg L^−1^ MF-NPs exposure) stained with NBT (to detect O_2_^∙−^). **d**, **e** Root maturation zone phenotype and relative ROS content in root matuaration zone with H_2_O_2_ stained with DAB. **f** Morphological changes of root hair on day 4 post inoculation with rhizobia. **g** Number of curled root hair (treatment number, n = 9)

The effect of MF-NP exposure on the ROS level in soybean roots was examined by tissue staining and localization of soybean root tips and root maturation zone. The roots were stained with either 3, 3-diaminobenzidine (DAB) or nitroblue tetrazolium (NBT) to observe H_2_O_2_ and O_2_^∙−^ production, respectively. Compared with the control, MF-NP-treated plants showed the accumulation of H_2_O_2_ and O_2_^∙−^ in the root tips (Fig. 4b, c) and root maturation zone (Fig. 4d, e). The degree of coloring was calculated with ImageJ software [31]. The content of H_2_O_2_ and O_2_^∙−^ in the MF-NPs treatment group was 6.52 and 2.08 times as high as that in the control group in the root tip, and 2.73 and 2.99 times in the root maturation zone. The detection results of total ROS content in root tips and root maturation zone were consistent with those described above (Additional file 1: Fig. S13).

### Evaluation of nodulation and nitrogen fixation efficiency

**Fig. 5:**
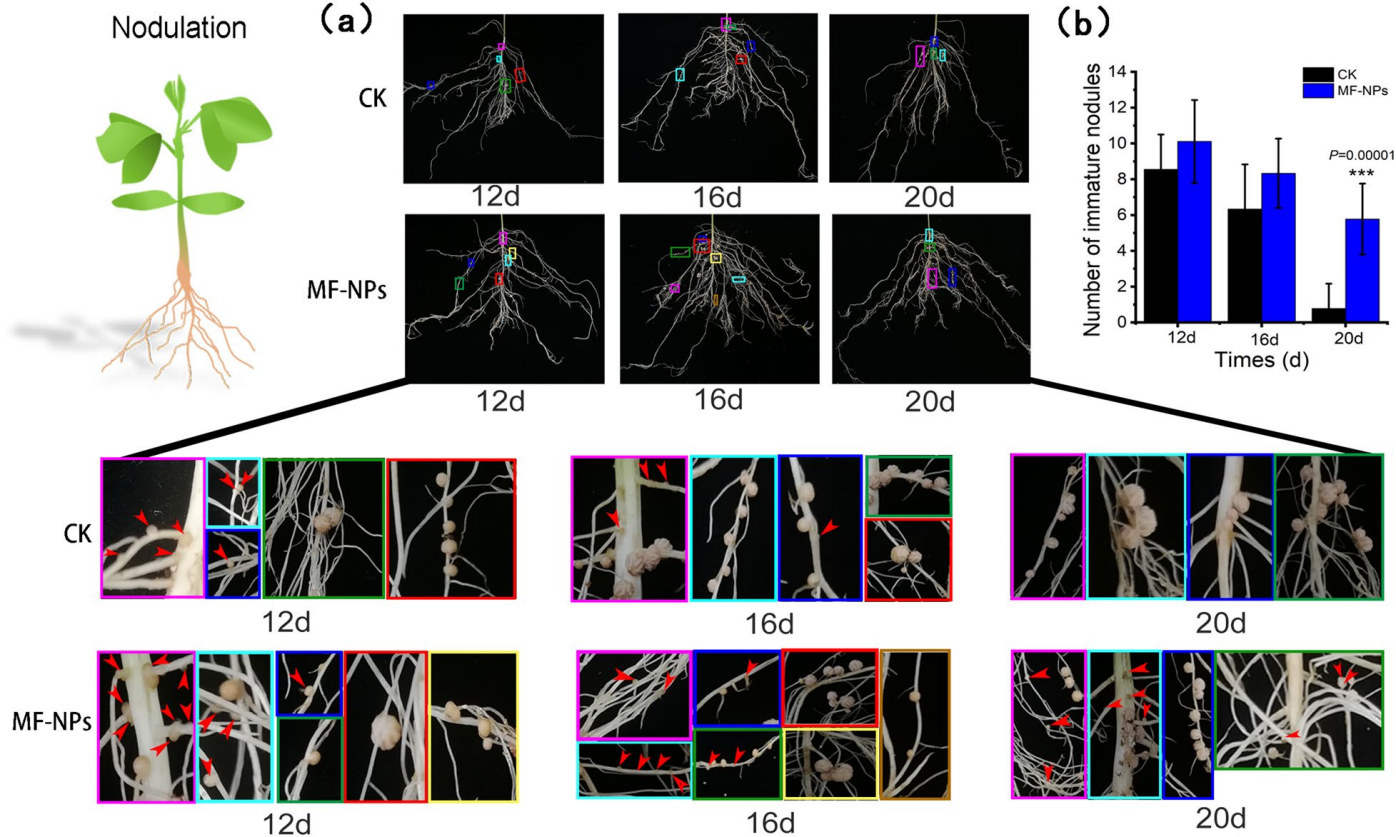
Early nodulation response to MF-NPs exposure. **a** Distribution of immature nodules at the various stage (on day 12 (early), 16 (middle), and 20 (late) post rhizobium inoculation, respectively) with/without MF-NPs exposure. **b** Number of nodules (n = 9)

Nodulation starts from the flavonoid-induced Rhizobium infection, leading to the expression of nodulation factor (Nod-F). In nodulation process, ROS first induces root hair curling, and then the development of infection lines and nodule primordia [36]. In this study, we counted the early curled root hairs. As shown in Fig. 4f and g, MF-NPs exposure increased the curling of root hairs in the maturation zone, which was 3.38 times of that in the control group, thus providing a prerequisite for the development and growth of mature nodules. With the further growth of nodules, early immature nodules visible to the naked eye were formed, and their number at different stages was used to locate the nodule development period. The difference in the distribution of immature nodules was observed between the control group and MF-NP treatment group during the 20-day growth period (Fig. 5a). The number of immature nodules in the 20-day growth period was found to be 7.43 times of that in the control group, indicating that the exposure to 10 mg L^−1^ MF-NPs could prolong the early development of the nodules, leading to a continuous increase in the number of nodules (Fig. 5b).

**Fig. 6:**
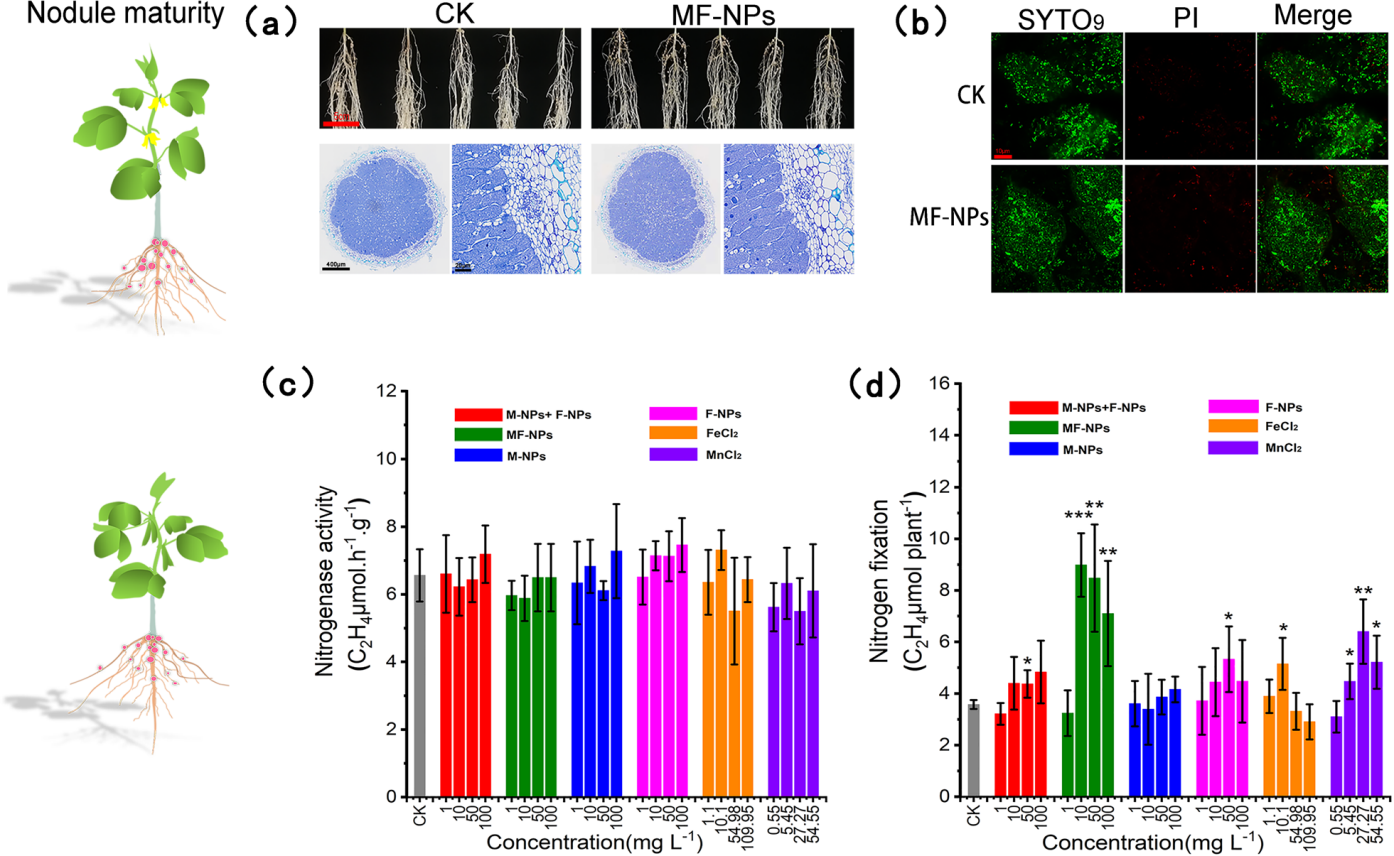
**a** Development of soybean root in 4 weeks post rhizobium inoculation with/without 10 mg L^−1^ MF-NPs exposure and the nodule microsection with toluidine blue staining. **b** Viability of symbiotic rhizobia evaluated by SYTO_9_. Green indicates all bacteria, and red after PI staining denotes dead bacteria. **c** Normalized nitrogenase activity (n = 5). **d** Individual plant nitrogen fixation efficiency (n = 5). *P < 0.05, **P < 0.01, and ***P < 0.001 indicate three different significance levels

Root phenotype and nodule development were used to evaluate the influence of MF-NP treatment on the growth and development of underground parts (Fig. 6a). Analysis of root morphology showed no obvious suppression effect of MF-NPs on root, and further analysis of the nodule section showed no obvious difference in the cell morphology and size between the infected area and the non-infected area, and the development of the transport tissue was normal. Fluorescence staining with SYTO_9_ and PI showed that the infected rhizobia in the cells of the nodule exhibited viability, and that the number of total infected rhizobia (green) and the dead rhizobia (red) in MF-NPs treatment group was comparable to that in the control group (Fig. 6b). These observations indicated that MF-NPs exposure had no obvious effect on the growth and development of soybean roots and nodules, and it could even increase root length.

The acetylene reduction method (ARM) was used to evaluate the nitrogenase activity in the mature nodules. All the treatments resulted in no significant difference in the nitrogenase activity per unit mass nodule, implying that the exposure to these nanomaterials would neither promote nitrogenase activity nor impose stress on the nitrogen fixation function of nodules (Fig. 6c). Furthermore, the total nitrogen fixation within 2 h in a single soybean plant (Fig. 6d) was evaluated by analyzing the reduction activity of the whole plant nodules. The 2.51-, 2.37-, 1.99-time increase in total nitrogen fixation under MF-NPs treatment (10 mg L^−1^, 50 mg L^−1^, and 100 mg L^−1^) and 1.79-time increase under MnCl_2_ (27.27 mg L^−1^) treatment, relative to the control group.

### Gene expression analysis

**Fig. 7:**
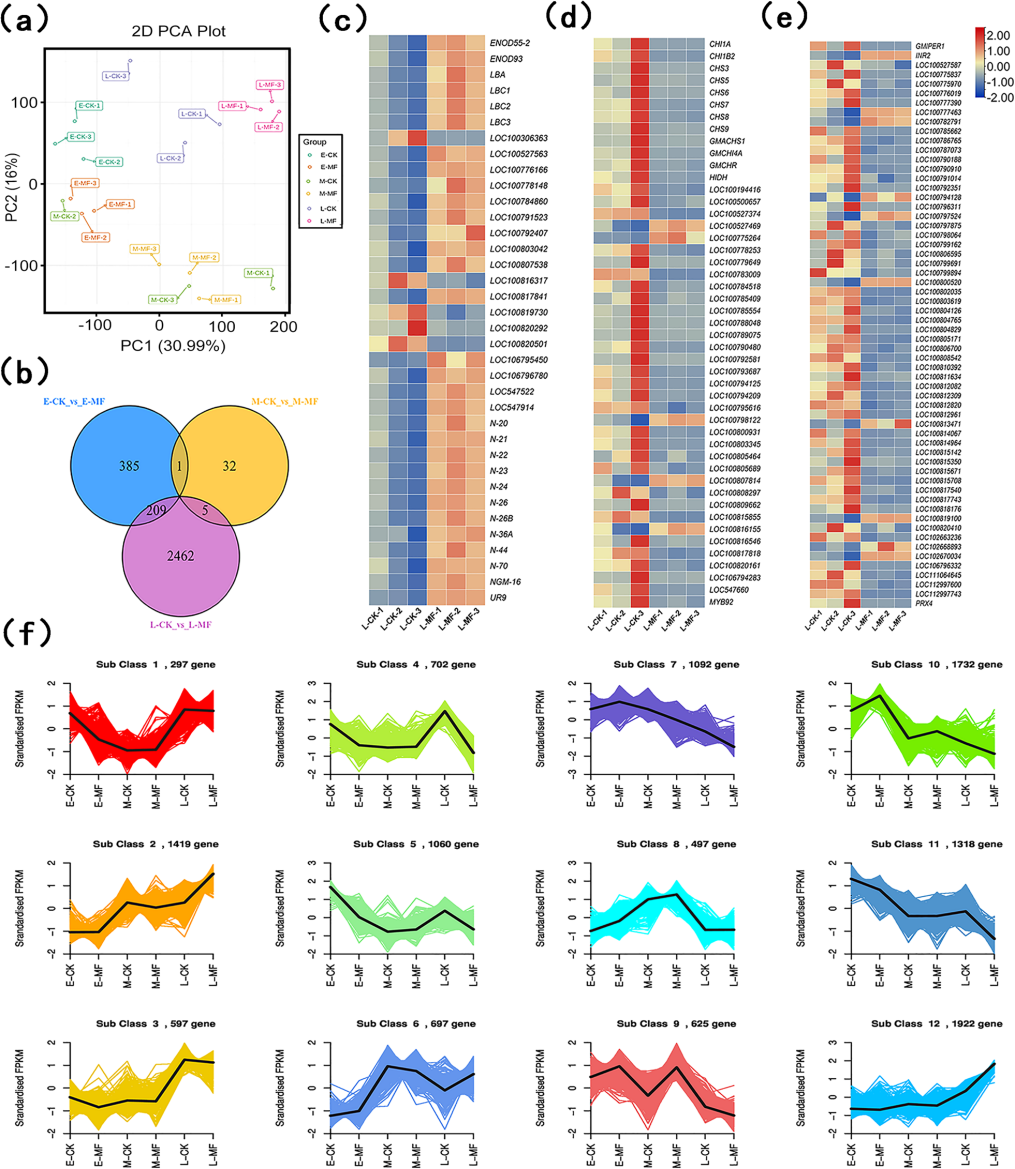
Global expression profiles of soybean genes at different nodulation stages. **a** Principal component analysis (PCA) of RNA-Seq data. **b** Venn diagram of E-CK (early stage), M-CK (middle stage), and L-CK (late stage) without NPs exposure. E-MF, M-MF, and L-MF indicate early-stage, middle-stage, and late-stage MF-NPs (10 mg L^−1^) exposure, respectively. **c**–**e** Relative expression heatmap of **c** Nod-R genes, **d** Fla-R genes, and **e** ROS-R genes. **f** K-means diagram of differential expressed genes (DEGs)

The mechanism by which MF-NPs exposure induced nodulation was investigated by transcriptome sequencing analysis of root samples from control and treatment groups at different stage (early stage, day 4; middle stage, day 8; and late stage, day 12 post inoculation). First, principal component analysis (PCA) was performed to determine the impacts of MF-NPs on soybean (Fig. 7a). A total of 595 DEGs were detected in the early stage with 100 up-regulated and 495 down-regulated, and 2676 DEGs in the late stage with 912 up-regulated and 1764 down-regulated. Hierarchical clustering analysis showed significant differences in different stages (Additional file 1: Figs. S14a, c and S15). However, only 38 DEGs (35 up-regulated and 3 down-regulated) were detected in the middle stage, indicating that MF-NPs exposure in the middle stage made little difference in the transcription level (Additional file 1: Fig. S15b). The Venn diagram showed the relatively few overlapping DEGs in three different stages (Fig. 7b).

The relationship between DEGs and nodulation metabolism was further explored by KEGG, NR, SwissProt, TrEMBL, KOG, GO and Pfam as a reference. The 5, 0, and 36 DEGs in the early, middle and late stages were found to be nodulation-related (Nod-R) with 2, 0, and 31 up-regulated, respectively (Fig. 7c). The late-stage DEGs were shown in Additional file 1: Table S2. The promotion effect of MF-NPs exposure on nodulation was mainly found in the early and late stages, especially in the late stage. We further analyzed flavonoid-related (Fla-R) DEGs (Fig. 7d, Additional file 1: Table S3) and ROS-related (ROS-R) DEGs (Fig. 7e, Additional file 1: Table S4) at late stage. Of 61 ROS-R DEGs, 51 down-regulated and 10 up-regulated. In total, 47 differential Fla-R DEGs were detected, of which 41 down-regulated and 6 up-regulated. In order to study the expression patterns of DEGs under different processing conditions, the FPKM of the DEGs was normalized, followed by K-means cluster analysis (Fig. 7f). Based on the expression patterns, Nod-R DEGs were clustered in class 12, and no significant difference between treatment group and control group in the early and middle stages, but a significant increase in the expression level of Nod-R DEGs was observed under MF-NPs exposure in the late stage. After optimization and clustering, the ROS-R DEGs were clustered into class 4, class 5, class 7, class 10, and class 11, all of which showed an opposite trend to class 12, which might be due to the feedback regulation triggered by the MF-NP-induced ROS increase. The Fla-R DEGs were mainly clustered into Class 4, Class 5, and Class 11, which was overlapped with the clustering of ROS-R, suggesting a synergistic relationship between these two types of DEGs. Our results were consistent with previous reports that expression of flavonoids could induce ROS expression [37, 38]. In addition, our results indicated that the expression patterns of Fla-R DEGs were completely opposite to those of Nod-R DEGs. Class 2 and class 3 showed the trend similar to that of Class12, indicating these 3 classes of DEGs might have synergistic effect, which remains to be further investigated.

**Fig. 8:**
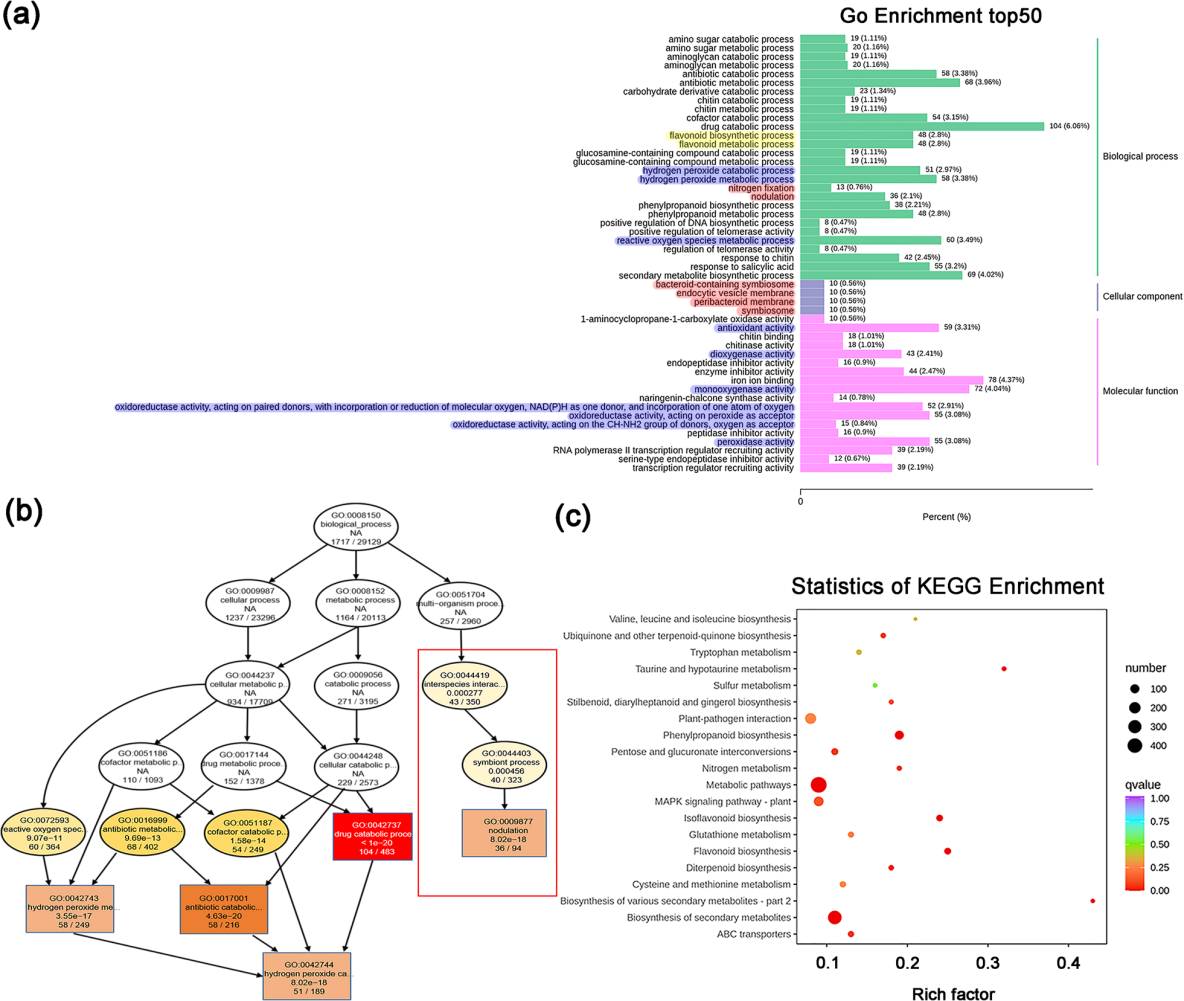
GO and KEGG enrichment analyses of DEGs identified from soybean. **a** GO enrichment histogram of top 50 GO function terms of DEGs (late stage). **b** Directed acyclic graph (DAG) of enriched GO terms (late stage). Each node represents a GO term, and the rectangle represents the top 5 enriched GO terms. The ellipse represents the included nodes. Different color shades represent the relative enrichment degree. The darker the color, the more significant the enrichment. The white color represents insignificant enrichment. The four rows of data for each node respectively represent the GO term ID, function, P value, and the number of DEGs /total number of genes in the GO term. **c** Top 20 significantly enriched KEGG pathways at late stage

GO enrichment analysis classified DEGs into biological process, cellular component and molecular function according to gene functions. Different genes in organisms exert biological functions through interactions. The 50 most significantly enriched GO-Terms were selected and classified according to GO database annotations. In the early stage (Additional file 1: Fig. S16), DEGs were mainly enriched in the pathways related to photosynthetic system inhibition and ROS metabolism. In the middle stage, DEGs were mainly enriched in the pathways (Additional file 1: Fig. S17) involved in cation transport. In the late stage (Fig. 8a), DEGs were mainly enriched in the pathways related to the secondary metabolism. Flavonoids (yellow box) serve as the initial inducers of legume nodulation. Our results indicated that the expression level of Fla-R genes was significantly reduced in the late stage, which might be due to the feedback regulation induced by the ROS increase in the early and middle stages. This was consistent with the expression of ROS-R genes in root samples (Fig. 8a). The microorganism-plant symbiosis system related to nodulation was significantly enhanced (as shown in Fig. 8a red box and Additional file 1: Fig. S18), which was specifically manifested as the enhancement in nodulation, nitrogen fixation, endocytic vesicle membrane, bacteroid-containing symbiosome, peribacteroid membrane, and other functions. The obtained GO terms were subjected to topGO directed acyclic graph (DAG) analysis. During symbiont process, the microorganism-plant interspecies interactions occurred nodulation upstream (red box in Fig. 8b and Additional file 1: Fig. S18). Changes in the metabolism of ROS were also reflected in multiple hierarchical pathways (Fig. 8b and Additional file 1: Fig. S19). The KEGG pathway analysis revealed the top 20 most significantly enriched pathways (Fig. 8c) with “biosynthesis of secondary metabolites” pathway exhibited the highest enrichment degree, which might be due to peroxide stress induction in soybean roots (Fig. 8c, Additional file 1: Fig. S20). KEGG pathway analysis indicated that “Flavonoid synthesis” pathway was significantly enriched, which was consistent with the results of GO enrichment analysis (Fig. 8c, Additional file 1: Fig. S21). KEGG pathway analysis also showed that glutathione metabolism pathway was significantly enriched, which might be due to the continuous increase in ROS, and this pathway enrichment contributed to the metabolic balance between root tissues and relieve oxidative stres (Fig. 8c and Additional file 1: Fig. S22). In addition, the enrichment of nitrogen metabolism pathway was observed, which might be explained by the increase in nitrogen fixation in some mature nodules in the late stage (Fig. 8c and Additional file 1: Fig. S23).

### MF-NPs-mediated AON response

**Fig. 9:**
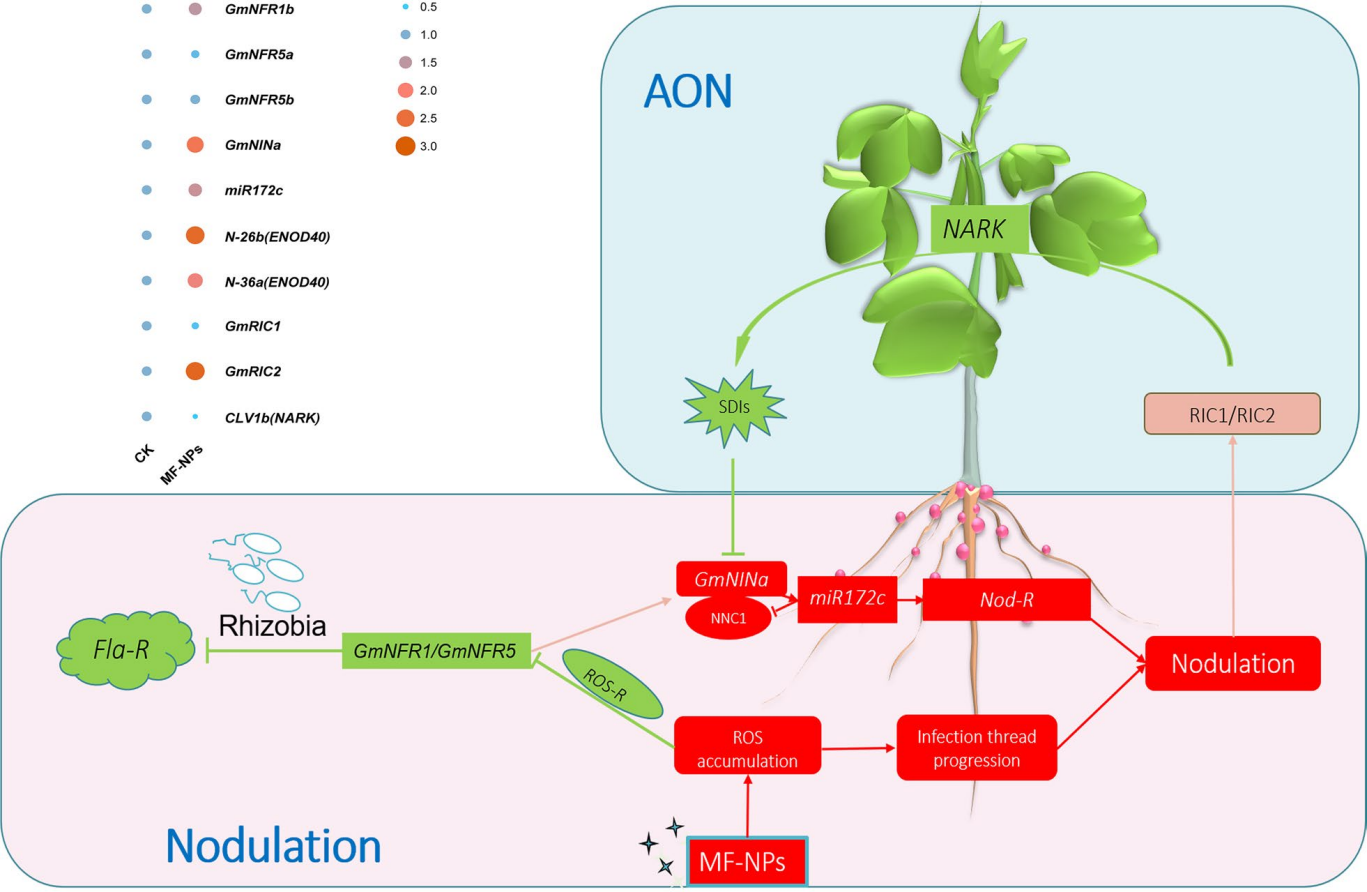
Model for exogenous ROS regulation of the balance between nodulation and AON. Related genes in the green box indicate down-regulated genes, and those in the red box indicate up-regulated genes

According to previous studies, the relevant autoregulation of nodulation (AON) pathway genes were identified [15, 17]. To evaluate the regulatory effect of MF-NPs exposure on the AON pathway, we analyzed the expression levels of the related genes, including *NFR1/NFR5* (nodulation factor), *GmNINa* (nodulation gene), *ENOD40s* (nodulation response gene), *NNC1* (nodule number control), *miR172c* (fine-tuning *rhizobium* infection and nodule organogenesis), *GmRIC1* and *GmRIC2* (specific CLAVATA/ESR-related (CLE) peptides in soybean responsible for producing root-derived nodulation), and *GmNARK* (nodule autoregulation receptor kinase) (Fig. 9). In the nodulation pathway, our study showed that the expression levels of Fla-R and ROS-R genes in MF-NPs exposure groups were overall down-regulated, while Nod-R genes showed a up-regulation expression trend. The expression of nodulation factors (*NFR1/5*) was low and not significantly different between MF-NPs exposure group and CK, which might be cause of the down-regulation of the ROS-R genes. In addition, *GmNINa*, *miR172c*, and *ENOD40s* all showed an up-regulation trend with significantly increasing ROS levels during this process. Therefore, the up-regulated expression of nodulation genes (*GmNINa*, *miR172c*, and *ENOD40s*) and the low expression of nodulation factors (*NFR1/5*) might be due to the increase in exogenous ROS. In the AON pathway, NARK made no obvious response to significant up-regulation of *GmRIC2* and *GmRIC1*expressions, which might be attributed to the inhibition of CLE peptides responsible for long-distance transportation. Therefore, MF-NPs exposure could enhance the expression of nodulation genes, meanwhile inhibiting the AON pathway, thereby achieving an increase in the number of nodules.

## Discussion

Divalent manganese ions have been reported to produce Fenton-like reactions, and they have a better hydroxyl radical induction effect in the FTR system [29, 39]. Although M-NPs, M-NPs+F-NPs, and FeCl_2_ showed strong H_2_O_2_ removal efficiency, most of their catalytic products was oxygen rather than ∙OH. Our data showed that FeCl_2_ had a high ∙OH generation efficiency, but ∙OH generation persistence was not satisfactory. Therefore, MF-NPs might be the best option for long-term ROS induction. In previous studies, MF-NPs have been widely used as an inducer of ∙OH originated from the FTR between Fe^2+^ and H_2_O_2_ and the Fenton-like reaction among Mn^2+^, Fe^2+^, andH_2_O_2_ in diatomic compounds [39]. The redox reaction between iron and manganese can further increase the yield of ∙OH, thus enriching the ROS type and content.

During plant growth, Mn ions participate in processes such as photosynthesis, respiration, protein synthesis, and hormone response [40]). Previous studies have shown that maize (*Zea mays*) growth is inhibited only under 200 mg kg^−1^ Mn exposure [41], whereas sunflower growth is not inhibited until Mn concentration reaches 5300 mg kg^−1^ [42]. Theoretically, exposure to 0–100 mg L^−1^ MF-NPs will not impose a serious stress on plant growth, thus the dwarfing of soybean plant is not caused by the stress of manganese ions. ROS stress has been reported to affect the plant root and height [43]. Therefore, in this study, the change of soybean plant height might result from ROS regulation. Our data showed that despite a significant plant height reduction, the total biomass of soybean plant was significantly increased, which can be attributed to the increase in the total nitrogen fixation efficiency of nodules in a single plant. However, this advantage disappeared under exposure to high concentrations of MF-NPs, suggesting an inhibitory effect of excessively high ROS stress on total nitrogen fixation. Our elemental composition analysis indicated that biomass was obviously higher in the system containing iron and manganese double ions than that in CK group, which was consistent with our assumption about the FTR between iron and manganese ions (Fig. 1b, Additional file 1: Table S1). Although the combination of M-NPs+F-NPs also contained iron and manganese ions, such a combination failed to exhibit a promoting effect, which might be due to the physical distance between the two ions. The MF-NPs exposure group exhibited a significant accumulation of nodule number and weight, which might be explained by the positive regulatory effect of ROS on the early nodulation system of soybean. Our results are consistent with one previous report [12].

Our data showed that the H_2_O_2_ and O_2_^∙−^ in the root were mainly derived from the induction of MF-NPs (Fig. 4a). Under MF-NPs exposure, H_2_O_2_ or O_2_^∙−^ can be converted into ∙OH through FTR to further enrich and enhance the ROS [44]. Previous studies have revealed that nanoparticles can significantly increase H_2_O_2_ and O_2_^∙−^ in plant roots, and the application of nanomaterials enhances plant resistance to the stresses such as drought, insect, and disease [45–47]. Therefore, nanomaterials conducive to enhancing plants’stress resistance need to be further developed and utilized.

The ROS regulatory gene *Rbohs* is used as a target gene to promote ROS yield, the overexpression of *Rbohs* can increase ROS yield, whereas RNA interference (RNAi) often decrease ROS yield [48]. In this study, under the optimal concentration (10 mg L^−1^) of MF-NPs exposure, *Rbohs* overexpression exhibited similar effect on the ROS generation to the pH-dependent FTR. Within an appropriate H_2_O_2_ concentration range (0.01–0.3%), the excessively acidic matrix exhibited significant inhibition on the soybean development, indicating that ∙OH indirectly inhibited the soybean growth through Fenton reaction, while high concentration of H_2_O_2_ (> 0.3%) directly inhibited root growth. Based on all above findings, it could be concluded that only a proper ROS concentration could induce an increase in the number and weight of nodules.

Nitrogenase can directly supply nitrogen nutrient for soybean, thus nitrogenase activity is used to evaluate nitrogen fixation of soybean. In our previous study, ROS was found to irreversibly damage nitrogenase activity [30]. In the present study, we did not observe a significant inhibition of unit nitrogenase activity by ROS, which might be due to the leghaemoglobin-supplied strong reducing conditions and MF-NPs exposure-induced ROS degradation [49]. In the early root development and early nodulation, the increasing evidence support that nodulation efficiency is highly dependent on ROS accumulation [50]. FTR provides a relatively high ROS in early nodulation, but the reaction between Fe^2+^ and Mn^2+^ tends to be complete when ROS is not needed in large amount during the nodule maturity stage. Therefore, the ROS exposure will not have an inhibitory effect on nitrogenase activity, resulting in the significant increase in nitrogen fixation efficiency of a single plant.

In this study, transcriptomics analysis confirmed the early experimental phenomena from the genetic level and metabolic pathways. In previous studies, the induction of carbon nanomaterials has been reported to increase the expression of the *Lotus japonicus* early nodulation genes [51], but the related action mechanism remains unclear. Our study provides evidence for the exploration of this mechanism. In the study of toxicity effect of engineered nanomaterials (ENM) (Ag, ZnO, and TiO_2_) on the legume *Medicago sativa* A17 [52], the early nodulation genes have been found to be significantly down-regulated, and the inhibition of nodulation under the ENM treatment might be primarily be due to abiotic stress induced by Zn ions. In non-legume rice, fluorescent silica (F-SiO_2_) ENM treatments have been revealed to increase biomass accumulation, and transcription analysis has unveiled that F-SiO_2_ ENM treatment could upregulate the expression of the genes related to glucose metabolism and carbon fixation [53]. These previous findings are in line with the expression patterns of the related genes in MF-NPs-exposed soybean in the middle stage post inoculation in our study. The combined treatment of titanium dioxide and carbon dioxide can enhance the antioxidant activity of rice, down-regulate the expression of the genes related to flavonoids and other secondary metabolites, thereby enhancing photosynthesis [54]. This is in accordance with our findings of the inhibitory effect of MF-NPs on the expression of flavonoid-related genes in this study.

## Supplementary Information

**Additional file 1: Fig. S1.** TEM images of (a) MF-NPs, (b) M-NPs, and (c) F-NPs. **Fig. S2.** Fourier transform infrared (FTIR) spectra of F-NPs, M-NPs and MF-NPs. **Fig. S3.** Comparison of the efficiency of oxygen production by different materials. **Fig.S4.** Ultraviolet scan curve of MB fading at different reaction time points in the presence of NPs and CMs. **Fig. S5.** Aboveground phenotypic images of soybean plants treated with different NPs. **Fig. S6.** Soybean plant height under exposure to different materials. *P < 0.05, **P < 0.01, and ***P < 0.001 respectively indicate 3 different significance levels. **Fig. S7.** Stem diameter of soybean plant under exposure to different materials. *P < 0.05, **P < 0.01, and ***P < 0.001 respectively indicate three different significance levels. **Fig. S8.** Phenotypic image of root system of soybean treated with different NPs. **Fig. S9.** Root length of soybean treated with different NPs. *P < 0.05, **P < 0.01, and ***P < 0.001 respectively indicate three different significance levels. **Fig. S10.** Total biomass of soybean under exposure to different NPs. *P < 0.05, **P < 0.01, and ***P < 0.001 respectively indicate three different significance levels. **Fig. S11.** Number of soybean nodules under exposure to different NPs. *P < 0.05, **P < 0.01, and ***P < 0.001 respectively indicate three different significance levels. **Fig. S12.** Weight of soybean nodules under exposure to different NPs. *P < 0.05, **P < 0.01, and ***P < 0.001 respectively indicate three different significance levels. **Fig.S13.** Quantitative analysis of total ROS in root. *P < 0.05, **P < 0.01, and ***P < 0.001 respectively indicate three different significance levels. **Fig. S14.** Number of differentially expressed genes (DEGs) detected in the three stages (early, middle, and late) post inoculation. Red indicates the significantly up-regulated DEGs. Green indicates the significantly down-regulated DEGs. Blue indicates DEGs with no significant expression change. **Fig. S15.** Heat map of cluster analysis of differentially expressed genes in the three different stages post inoculation. **Fig. S16.** GO enrichment histogram of differentially expressed genes (DEGs, early stage). **Fig. S17.** GO enrichment histogram of differentially expressed genes (DRGs, middle stage). **Fig. S18.** Directed acyclic graph (DAG) of enriched GO terms (late stage). **Fig. S19.** Directed acyclic graph (DAG) of GO enrichment analysis of ROS metabolism-related genes (late stage). **Fig. S20.** KEGG pathway of biosynthesis of various secondary metabolites—part 2 in response to MF-NPs treatment. **Fig. S21.** KEGG pathway of flavonoid biosynthesis in response to MF-NPs treatment. **Fig. S22.** KEGG pathway of glutathione metabolism in response to MF-NPs treatment. **Fig. S23.** Kyoto Encyclopedia of Genes and Genomes (KEGG) pathway of nitrogen metabolism in response to MF-NPs treatment. **Fig. S24.** Standard curve of nitrogenase activity. **Table S1.** Elemental content of NPs. **Table S2.** Expression of nodulation related (Nod-R) genes in the roots in response to MF-NPs treatment. **Table S3.** Expression of flavonoid related (Fla-R) genes in the roots in response to MF-NPs treatment. **Table S4.** Expression of reactive oxygen species related (ROS-R) genes in roots in response to MF-NPs treatment.

## Data Availability

The datasets analysed in the current study are available from the corresponding author on reasonable request.

## References

[1] Rogers C, Oldroyd GE (2014). Synthetic biology approaches to engineering the nitrogen symbiosis in cereals. J Exp Bot.

[2] Sá AGA, Moreno YMF, Carciofi BAM (2020). Plant proteins as high-quality nutritional source for human diet. Trends Food Sci Technol.

[3] Lowry GV, Avellan A, Gilbertson LM (2019). Opportunities and challenges for nanotechnology in the agri-tech revolution. Nat nanotechnol.

[4] Pulizzi F (2021). The rise of nanoagrochemicals. Nat nanotechnol.

[5] Kah M, Kookana RS, Gogos A, Bucheli TD (2018). A critical evaluation of nanopesticides and nanofertilizers against their conventional analogues. Nat Nanotechnol.

[6] Mittler R, Vanderauwera S, Suzuki N, Miller G, Tognetti VB, Vandepoele K, Gollery M, Shulaev V, Van Breusegem F (2011). ROS signaling: the new wave?. Trends Plant Sci.

[7] Puppo A, Pauly N, Boscari A, Mandon K, Brouquisse R (2013). Hydrogen peroxide and nitric oxide: key regulators of the Legume*-Rhizobium* and mycorrhizal symbioses. Antioxid Redox Signal.

[8] Cardenas L, Martinez A, Sanchez F, Quinto C (2008). Fast, transient and specific intracellular ROS changes in living root hair cells responding to Nod factors (NFs). Plant J.

[9] Shaw SL, Long SR (2003). Nod factor inhibition of reactive oxygen efflux in a host legume. Plant Physiol.

[10] Marino D, Dunand C, Puppo A, Pauly N (2012). A burst of plant NADPH oxidases. Trends Plant Sci.

[11] Marino D, Andrio E, Danchin EG, Oger E, Gucciardo S, Lambert A, Puppo A, Pauly N (2011). A *Medicago truncatula* NADPH oxidase is involved in symbiotic nodule functioning. New Phytol.

[12] Arthikala MK, Sanchez-Lopez R, Nava N, Santana O, Cardenas L, Quinto C (2014). *RbohB*, a *Phaseolus vulgaris* NADPH oxidase gene, enhances symbiosome number, bacteroid size, and nitrogen fixation in nodules and impairs mycorrhizal colonization. New Phytol.

[13] Carrasco-Castilla J, Ortega-Ortega Y, Jáuregui-Zúñiga D, Juárez-Verdayes MA, Arthikala M-K, Monroy-Morales E, Nava N, Santana O, Sánchez-López R, Quinto C (2018). Down-regulation of a *Phaseolus vulgaris* annexin impairs rhizobial infection and nodulation. Environ Exp Bot.

[14] Breakspear A, Liu C, Roy S, Stacey N, Rogers C, Trick M, Morieri G, Mysore KS, Wen J, Oldroyd GE (2014). The root hair "infectome" of *Medicago truncatula* uncovers changes in cell cycle genes and reveals a requirement for Auxin signaling in rhizobial infection. Plant Cell.

[15] Wang L, Sun Z, Su C, Wang Y, Yan Q, Chen J, Ott T, Li X (2019). A GmNINa-miR172c-NNC1 regulatory network coordinates the nodulation and autoregulation of nodulation pathways in soybean. Mol Plant.

[16] Mortier V, Den Herder G, Whitford R, Van de Velde W, Rombauts S, D'Haeseleer K, Holsters M, Goormachtig S (2010). CLE peptides control *Medicago truncatula* nodulation locally and systemically. Plant Physiol.

[17] Searle IR, Men AE, Laniya TS, Buzas DM, Ormaetxe II, Carroll BJ, Gresshoff PM (2003). Long-distance signaling in nodulation directed by a CLAVATA1-like receptor kinase. Science.

[18] Yu J, Zhao F, Gao W, Yang X, Ju Y, Zhao L, Guo W, Xie J, Liang XJ, Tao X (2019). Magnetic reactive oxygen species nanoreactor for switchable magnetic resonance imaging guided cancer therapy based on pH-sensitive Fe_5_C_2_@Fe_3_O_4_ nanoparticles. ACS Nano.

[19] Lin LS, Huang T, Song J, Ou XY, Wang Z, Deng H, Tian R, Liu Y, Wang JF, Liu Y (2019). Synthesis of copper peroxide nanodots for H_2_O_2_ self-supplying chemodynamic therapy. J Am Chem Soc.

[20] Yang Z, Qian J, Yu A, Pan B (2019). Singlet oxygen mediated iron-based Fenton-like catalysis under nanoconfinement. Proc Natl Acad Sci USA.

[21] Li X, Wang J, Rykov AI, Sharma VK, Wei H, Jin C, Liu X, Li M, Yu S, Sun C (2015). Prussian blue/TiO_2_ nanocomposites as a heterogeneous photo-Fenton catalyst for degradation of organic pollutants in water. Catal Sci Technol.

[22] Li X, Wang Z, Zhang B, Rykov AI, Ahmed MA, Wang J (2016). Fe_x_Co_3_−_x_O_4_ nanocages derived from nanoscale metal–organic frameworks for removal of bisphenol A by activation of peroxymonosulfate. Appl Cata B.

[23] Yang J, Zhang Y, Zeng D, Zhang B, Hassan M, Li P, Qi C, He Y (2020). Enhanced catalytic activation of photo-Fenton process by Cu_0.5_Mn_0.5_Fe_2_O_4_ for effective removal of organic contaminants. Chemosphere.

[24] Du Z, Li K, Zhou S, Liu X, Yu Y, Zhang Y, He Y, Zhang Y (2020). Degradation of ofloxacin with heterogeneous photo-Fenton catalyzed by biogenic Fe-Mn oxides. Chem Eng J.

[25] Yim B, Chock PB, Stadtman ER (1990). Manganese(II)-bicarbonate-mediated catalytic activity for hydrogen peroxide dismutation and amino acid oxidation: detection of free radical intermediates. Proc Natl Acad Sci USA.

[26] Qi C, Liu X, Ma J, Lin C, Li X, Zhang H (2016). Activation of peroxymonosulfate by base: Implications for the degradation of organic pollutants. Chemosphere.

[27] Fan J, Qin H, Jiang S (2019). Mn-doped g-C_3_N_4_ composite to activate peroxymonosulfate for acetaminophen degradation: the role of superoxide anion and singlet oxygen. Chem Eng J.

[28] Costa RC, Lelis MF, Oliveira LC, Fabris JD, Ardisson JD, Rios RR, Silva CN, Lago RM (2006). Novel active heterogeneous Fenton system based on Fe_3_-xMxO_4_ (Fe Co, Mn, Ni): the role of M^2+^ species on the reactivity towards H_2_O_2_ reactions. J Hazard Mater.

[29] Sahoo B, Sahu SK, Nayak S, Dhara D, Pramanik P (2012). Fabrication of magnetic mesoporous manganese ferrite nanocomposites as efficient catalyst for degradation of dye pollutants. Catal Sci Technol.

[30] Ma J, Song Z, Yang J, Wang Y, Han H (2021). Cobalt ferrite nanozyme for efficient symbiotic nitrogen fixation via regulating reactive oxygen metabolism. Environ Sci Nano.

[31] Kong X, Tian H, Yu Q, Zhang F, Wang R, Gao S, Xu W, Liu J, Shani E, Fu C (2018). PHB3 maintains root stem cell niche identity through ROS-responsive AP2/ERF transcription factors in arabidopsis. Cell Rep.

[32] Vega-Vásquez P, Mosier NS, Irudayaraj J (2021). Hormesis-inducing essential oil nanodelivery system protects plants against broad host-range necrotrophs. ACS Nano.

[33] Gao L, Liu Z, Yang Z, Cao L, Feng C, Chu M, Tang J (2020). Synthesis and magnetism property of manganese ferrite MnFe_2_O_4_ by selective reduction and oxidization roasting process. Appl Surf Sci.

[34] Long NV, Yang Y, Teranishi T, Thi CM, Cao Y, Nogami M (2015). Synthesis and magnetism of hierarchical iron oxide particles. Mater Des.

[35] Thota S, Kashyap SC, Sharma SK, Reddy VR (2016). Cation distribution in Ni-substituted Mn_0.5_Zn_0.5_Fe_2_O_4_ nanoparticles: a Raman, Mössbauer, X-ray diffraction and electron spectroscopy study. Mater Sci Eng B.

[36] Esseling JJ, Lhuissier FGP, Emons AMC (2003). Nod factor-induced root hair curling: continuous polar growth towards the point of nod factor application. Plant Physiol.

[37] Downie JA (2014). Legume nodulation. Curr Biol.

[38] Mazars C, Thuleau P, Lamotte O, Bourque S (2010). Cross-talk between ROS and calcium in regulation of nuclear activities. Mol Plant.

[39] Zhou Y, Xiao B, Liu SQ, Meng Z, Chen ZG, Zou CY, Liu CB, Chen F, Zhou X (2016). Photo-Fenton degradation of ammonia via a manganese–iron double-active component catalyst of graphene–manganese ferrite under visible light. Chem Eng J.

[40] Alejandro S, Holler S, Meier B, Peiter E (2020). Manganese in plants: from acquisition to subcellular allocation. Front Plant Sci.

[41] Schulte E, Kelling K. Soil and applied manganese: understanding plant nutrients. A2526 Madison, WI, USA: University of Wisconsin-Madison and University of Wisconsin-Extension;Cooperative Extension 1999.

[42] Shao JF, Yamaji N, Liu XW, Yokosho K, Shen RF, Ma JF (2018). Preferential distribution of boron to developing tissues is mediated by the intrinsic protein OsNIP3. Plant Physiol.

[43] Sun XD, Yuan XZ, Jia Y, Feng LJ, Zhu FP, Dong SS, Liu J, Kong X, Tian H, Duan JL (2020). Differentially charged nanoplastics demonstrate distinct accumulation in *Arabidopsis thaliana*. Nat Nanotechnol.

[44] Kim J, Kim HY, Song SY, Go SH, Sohn HS, Baik S, Soh M, Kim K, Kim D, Kim HC (2019). Synergistic oxygen generation and reactive oxygen species scavenging by manganese ferrite/ceria co-decorated nanoparticles for rheumatoid arthritis treatment. ACS Nano.

[45] El-Shetehy M, Moradi A, Maceroni M, Reinhardt D, Petri-Fink A, Rothen-Rutishauser B, Mauch F, Schwab F (2021). Silica nanoparticles enhance disease resistance in *Arabidopsis* plants. Nat Nanotechnol.

[46] Guerriero G, Sutera FM, Torabi-Pour N, Renaut J, Hausman JF, Berni R, Pennington HC, Welsh M, Dehsorkhi A, Zancan LR (2021). Phyto-Courier, A silicon particle-based nano-biostimulant: evidence from cannabis sativa exposed to salinity. ACS Nano.

[47] Ma C, Borgatta J, Hudson BG, Tamijani AA, Torre-Roche RDL, Zuverza-Mena N, Shen Y, Elmer W, Xing B, Mason SE (2020). Advanced material modulation of nutritional and phytohormone status alleviates damage from soybean sudden death syndrome. Nat nanotechnol.

[48] Montiel J, Nava N, Cardenas L, Sanchez-Lopez R, Arthikala MK, Santana O, Sanchez F, Quinto C (2012). A *Phaseolus vulgaris* NADPH oxidase gene is required for root infection by Rhizobia. Plant Cell Physiol.

[49] Du HY, Chen CM, Yu GH, Polizzotto ML, Sun FS, Kuzyakov Y (2020). An iron-dependent burst of hydroxyl radicals stimulates straw decomposition and CO_2_ emission from soil hotspots: consequences of Fenton or Fenton-like reactions. Geoderma.

[50] Roychowdhury R, Choudhury S, Hasanuzzaman M, Srivastava S: Sustainable Agriculture in the Era of Climate Change; 2020.

[51] Yuan Z, Zhang Z, Wang X, Li L, Cai K, Han H (2017). Novel impacts of functionalized multi-walled carbon nanotubes in plants: promotion of nodulation and nitrogenase activity in the rhizobium-legume system. Nanoscale.

[52] Chen C, Unrine JM, Judy JD, Lewis RW, Guo J, McNear DH, Tsyusko OV (2015). Toxicogenomic responses of the model legume *Medicago truncatula* to aged biosolids containing a mixture of nanomaterials (TiO_2_, Ag, and ZnO) from a pilot wastewater treatment plant. Environ Sci Technol.

[53] Cheng BX, Chen FR, Wang CX, Liu XF, Yue L, Cao XS, Wang ZY, Xing BS (2021). The molecular mechanisms of silica nanomaterials enhancing the rice (*Oryza sativa* L.) resistance to planthoppers (*Nilaparvata lugens Stal*). Sci Total Environ.

[54] Xu ML, Zhu YG, Gu KH, Zhu JG, Yin Y, Ji R, Du WC, Guo HY (2019). Transcriptome reveals the rice response to elevated free air CO_2_ concentration and TiO_2_ nanoparticles. Environ Sci Technol.

