# Supplementary material for: Novel approach to enhance Bradyrhizobium diazoefficiens nodulation through continuous induction of ROS by manganese ferrite nanomaterials in soybean
Source: J Nanobiotechnology. 2022 Mar 31;20:168. doi: 10.1186/s12951-022-01372-2 (PMC8973989; doi:10.1186/s12951-022-01372-2)
Supplement: Supplementary file 1 — Additional file 1: Fig. S1. TEM images of (a) MF-NPs, (b) M-NPs, and (c) F-NPs. Fig. S2. Fourier transform infrared (FTIR) spectra of F-NPs, M-NPs and MF-NPs. Fig. S3. Comparison of the efficiency of oxygen production by different materials. Fig.S4. Ultraviolet scan curve of MB fading at different reaction time points in the presence of NPs and CMs. Fig. S5. Aboveground phenotypic images of soybean plants treated with different NPs. Fig. S6. Soybean plant height under exposure to different materials. *P < 0.05, **P < 0.01, and ***P < 0.001 respectively indicate 3 different significance levels. Fig. S7. Stem diameter of soybean plant under exposure to different materials. *P < 0.05, **P < 0.01, and ***P < 0.001 respectively indicate three different significance levels. Fig. S8. Phenotypic image of root system of soybean treated with different NPs. Fig. S9. Root length of soybean treated with different NPs. *P < 0.05, **P < 0.01, and ***P < 0.001 respectively indicate three different significance levels. Fig. S10. Total biomass of soybean under exposure to different NPs. *P < 0.05, **P < 0.01, and ***P < 0.001 respectively indicate three different significance levels. Fig. S11. Number of soybean nodules under exposure to different NPs. *P < 0.05, **P < 0.01, and ***P < 0.001 respectively indicate three different significance levels. Fig. S12. Weight of soybean nodules under exposure to different NPs. *P < 0.05, **P < 0.01, and ***P < 0.001 respectively indicate three different significance levels. Fig.S13. Quantitative analysis of total ROS in root. *P < 0.05, **P < 0.01, and ***P < 0.001 respectively indicate three different significance levels. Fig. S14. Number of differentially expressed genes (DEGs) detected in the three stages (early, middle, and late) post inoculation. Red indicates the significantly up-regulated DEGs. Green indicates the significantly down-regulated DEGs. Blue indicates DEGs with no significant expression change. Fig. S15. Heat map of cluster a [file 12951_2022_1372_MOESM1_ESM.docx]

**Novel approach to enhance *Bradyrhizobium diazoefficiens* nodulation through continuous induction of ROS by manganese ferrite nanomaterials in soybean**

Jun Ma ^1^, Yi Zhou ^1^, Jiaying Li ^1^, Zhiyong Song^2*^ and Heyou Han ^1, 2*^

^1^State Key Laboratory of Agricultural Microbiology, College of Life Science and Technology, Huazhong Agricultural University, No. 1Shizishan Street, Hongshan District, Wuhan, Hubei 430070, China.

^2^State Key Laboratory of Agricultural Microbiology, College of Science, Huazhong Agricultural University, No. 1 Shizishan Street, Hongshan District, Wuhan, Hubei 430070, China.

* Corresponding authors :

E-mail addresses: songzhiyong@mail.hzau.edu.cn

E-mail addresses: hyhan@mail.hzau.edu.cn

**Material Characterization.**

MF-NPs (Purity: 99.5%, APS: 40 nm) and M-NPs (Purity: 99.9%, APS: 50 nm,) were purchased from Shanghai Bike New Material Technology Co., Ltd.（China; F-NPs (Purity: 98.0%, APS: 50 nm) from Aladdin Reagent Co., Ltd. (Shanghai, China); FeCl_2_ and MnCl_2_ (analytical purity) from Sinopharm Chemical Reagent Co., Ltd. (Shanghai, China). The properties of these materials were further determined by dissolving them in pure water and ultrasonication for 20 min to determine their particle size, electric potential and infrared spectra. The same samples were also analyzed by projection electron microscopy and scanning electron microscopy. SEM analysis was performed using a Zeiss Sigma300 (with Gemini lens) microscope operating at 100 Kv. The element valence of MF-NPs was analyzed by X-ray photoelectron spectroscopy (XPS) using an Thermo ESCALAB 250XI, and their hydrodynamic sizes and electric potential were measured using a dynamic light scattering instrument (Malvern).

**O_2_ Generation Assay.**

The O_2_ generation was tested as previously reported with some modifications.[1] Briefly, a total of 1M H_2_O_2_ was mixed separately with MF-NPs, F-NPs, M-NPs, F-NPs+ M-NPs, MnCl_2_ or FeCl_2_ in 20 mL of PBS at room temperature, followed by measuring the O_2_ concentration every 5 min using a dissolved oxygen meter (JPBJ-608). To observe O_2_ bubbles in an Eppendorf tube, 1M of H_2_O_2_ was mixed separately with MF-NPs, F-NPs, M-NPs, F-NPs+ M-NPs, MnCl_2_ and FeCl_2_ and monitored until 30 min.

**Search for related functional genes**

KEGG（Kyoto Encyclopedia of Genes and Genomes）, NR（Non-Redundant Protein Sequence Database）, SwissProt, TrEMBL, KOG (eukaryotic orthologous groups), GO (gene ontology) and Pfam（the protein families database）database were used to search for related DEGs, the specific use method is described in the literature reports[2-7].

**Total ROS detection**

The total ROS was detected by measuring the green fluorescent intensity of the cell-permeable fluorogenic dye 2’,7’-dichlorofluorescin diacetate (DCFH-DA). Briefly, a certain amount of fresh root was weighed into a centrifuge tube, followed by adding DCFH-DA and grinding into homogenate. Finally, the solution was vortexed and centrifuged, followed by measuring the fluorescence intensity. DCFH-DA was excited at 488 nm and emitted at 525 nm.


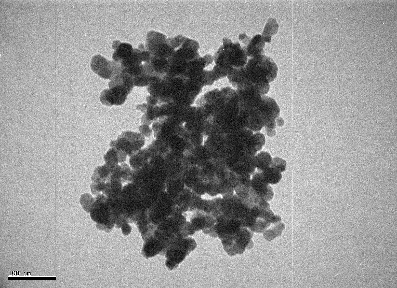

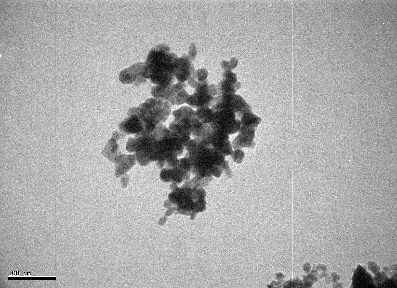

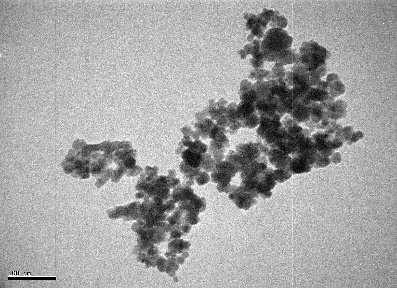


(a) （b） (c)

**Fig. S1** TEM images of (a) MF-NPs, (b) M-NPs, and (c) F-NPs.


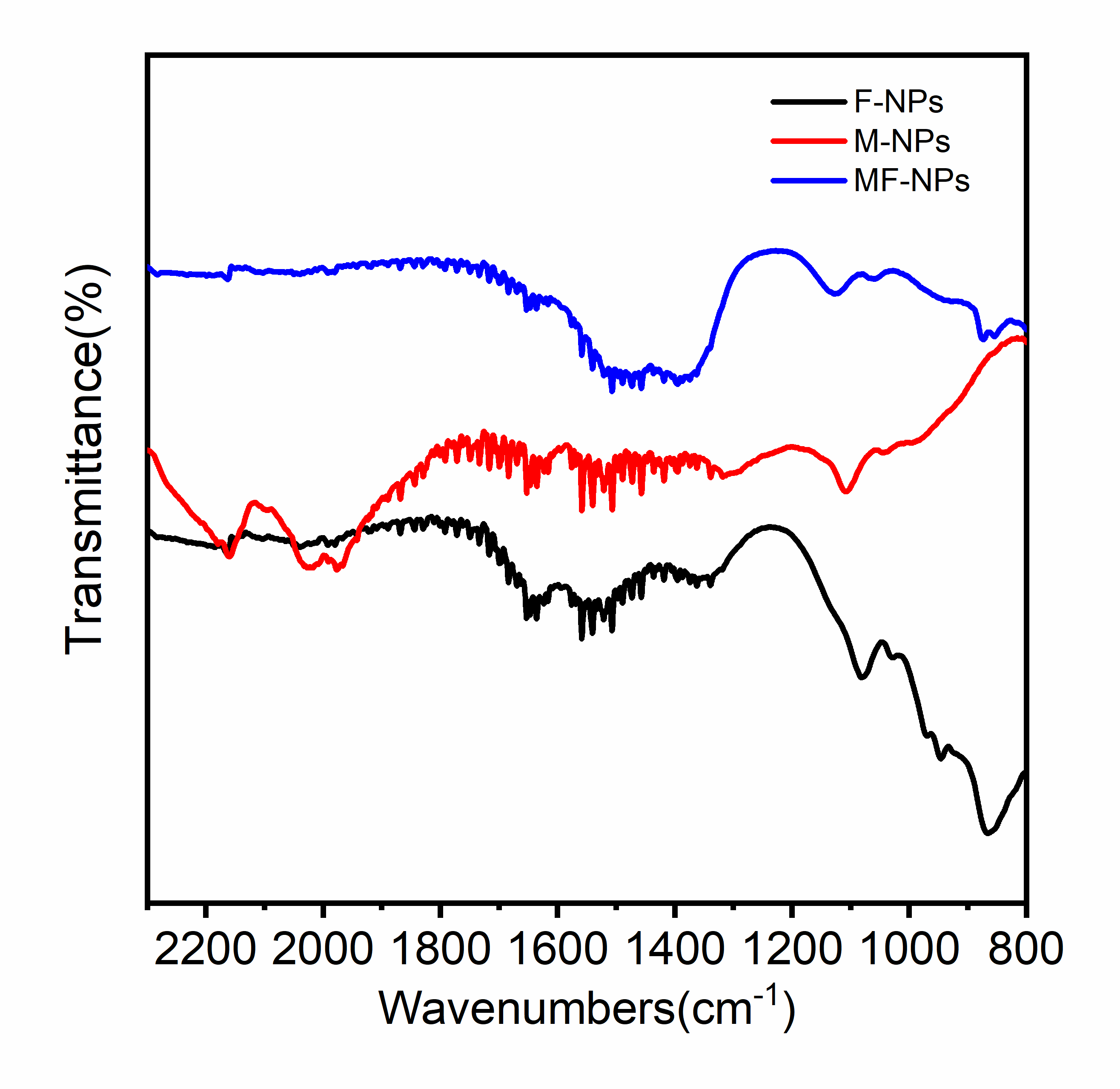


**Fig. S2** Fourier transform infrared (FTIR) spectra of F-NPs, M-NPs and MF-NPs.


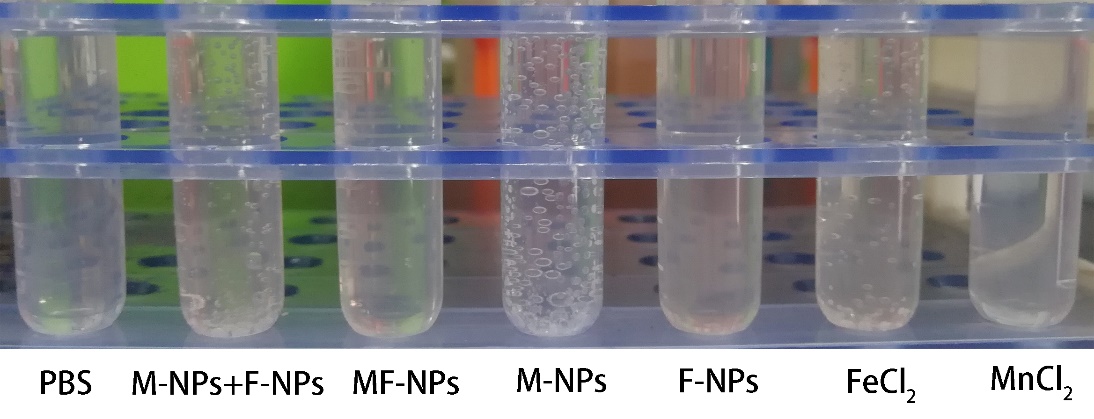


**Fig. S3** Comparison of the efficiency of oxygen production induced by different materials.


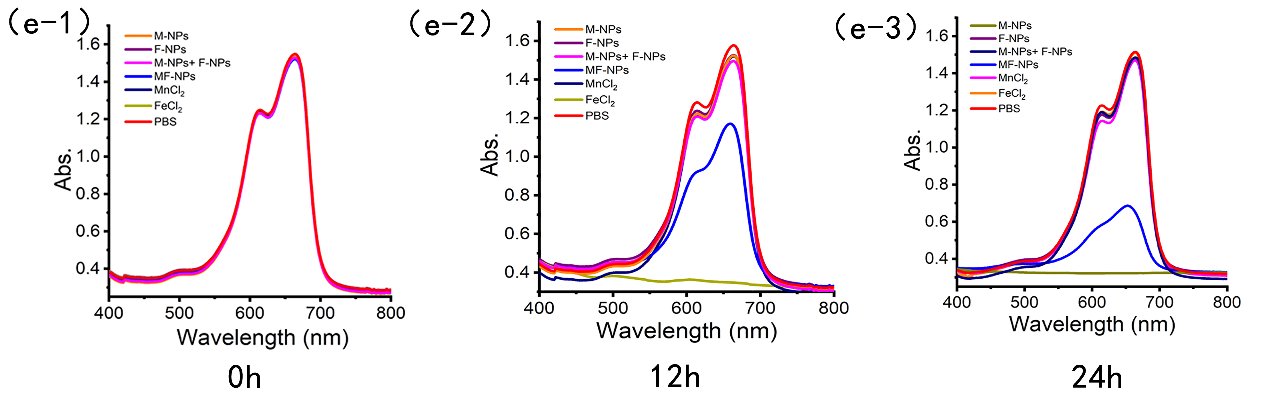


**Fig.S4** Ultraviolet scan curve of MB fading at different reaction time points in the presence of NPs and CMs.


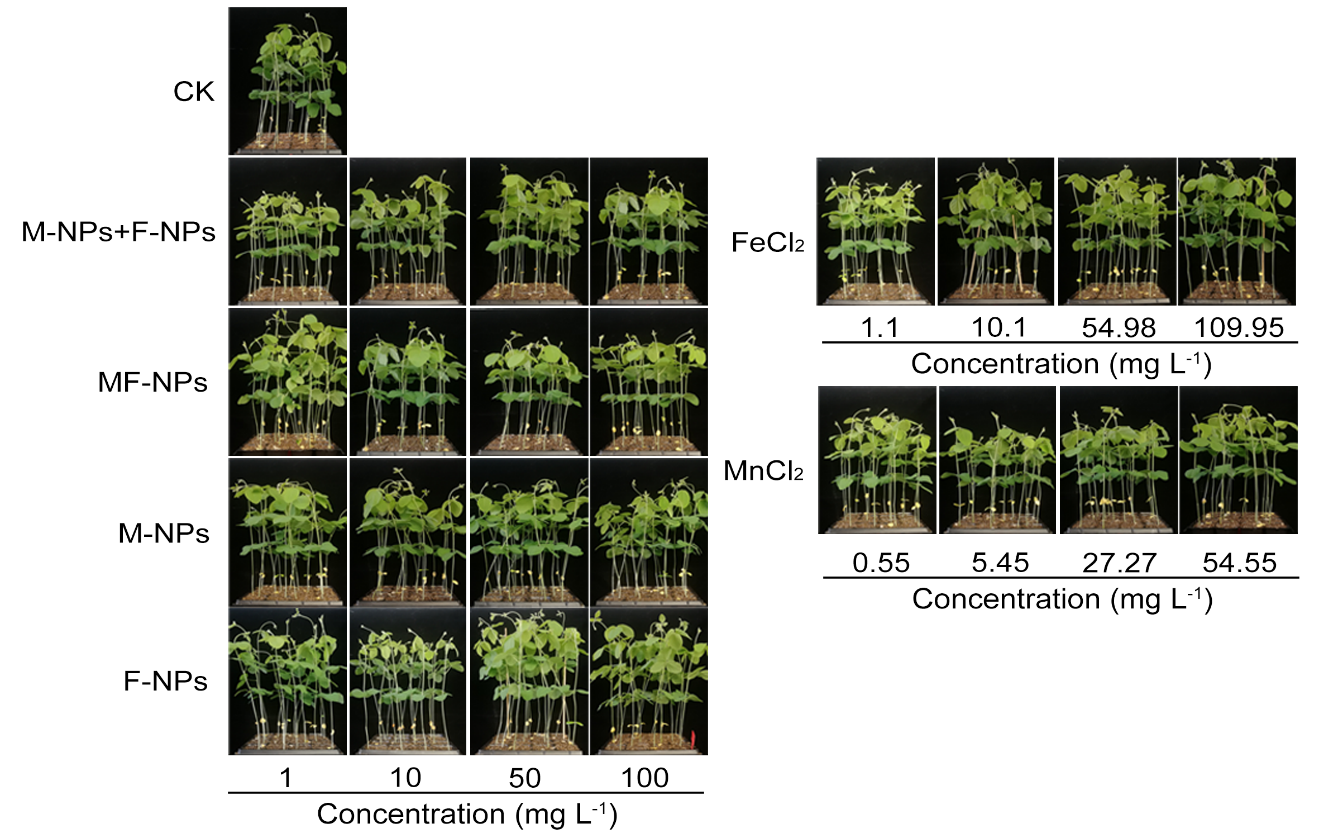


**Fig. S5** Aboveground phenotypic images of soybean matrix treated with different NPs.





**Fig. S6** Plant height of soybean under exposure to different materials. The significance levels were determined at *P < 0.05, **P < 0.01, and ***P < 0.001.





**Fig. S7** Stem diameter of soybean under exposure to different materials. The significance levels were determined at *P < 0.05, **P < 0.01, and ***P < 0.001.

**
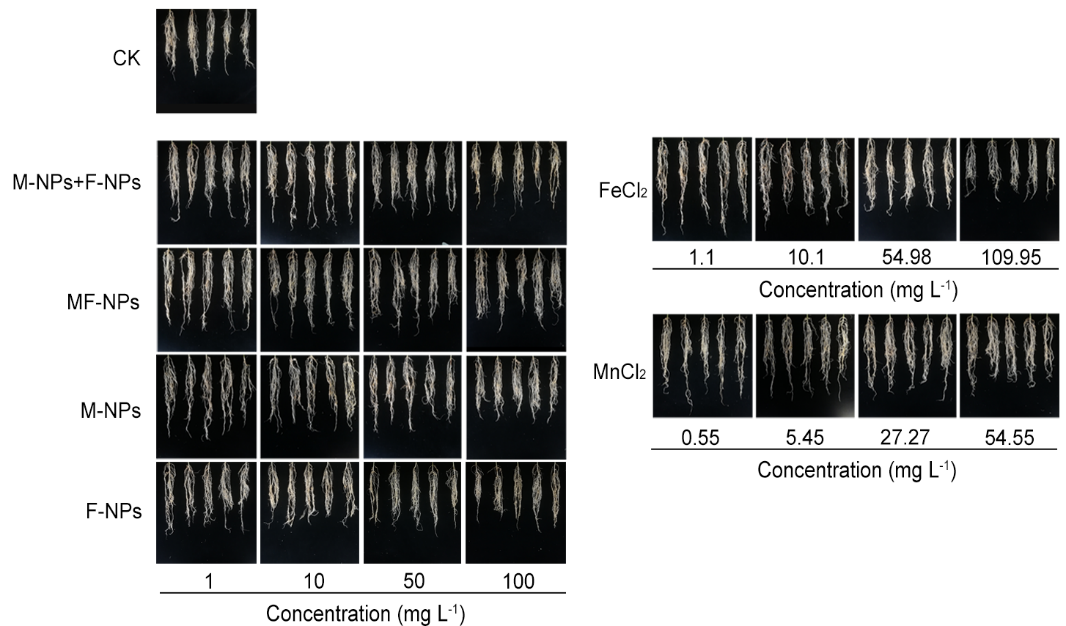
**

**Fig. S8** Phenotypic image of root system of soybean matrix treated with different NPs.





**Fig. S9** Root length of soybean matrix treated with different NPs. The significance levels were determined at *P < 0.05, **P < 0.01, and ***P < 0.001.





**Fig. S10** Total biomass of soybean under exposure to different NPs. The significance levels were determined at *P < 0.05, **P < 0.01, and ***P < 0.001.





**Fig. S11** Number of soybean nodules under exposure to different NPs. The significance levels were determined at *P < 0.05, **P < 0.01, and ***P < 0.001.





**Fig. S12** Weight of soybean nodules under exposure to different NPs. The significance levels were determined at *P < 0.05, **P < 0.01, and ***P < 0.001.





**Fig.S13** Quantitative analysis of total ROS in root (n=5).


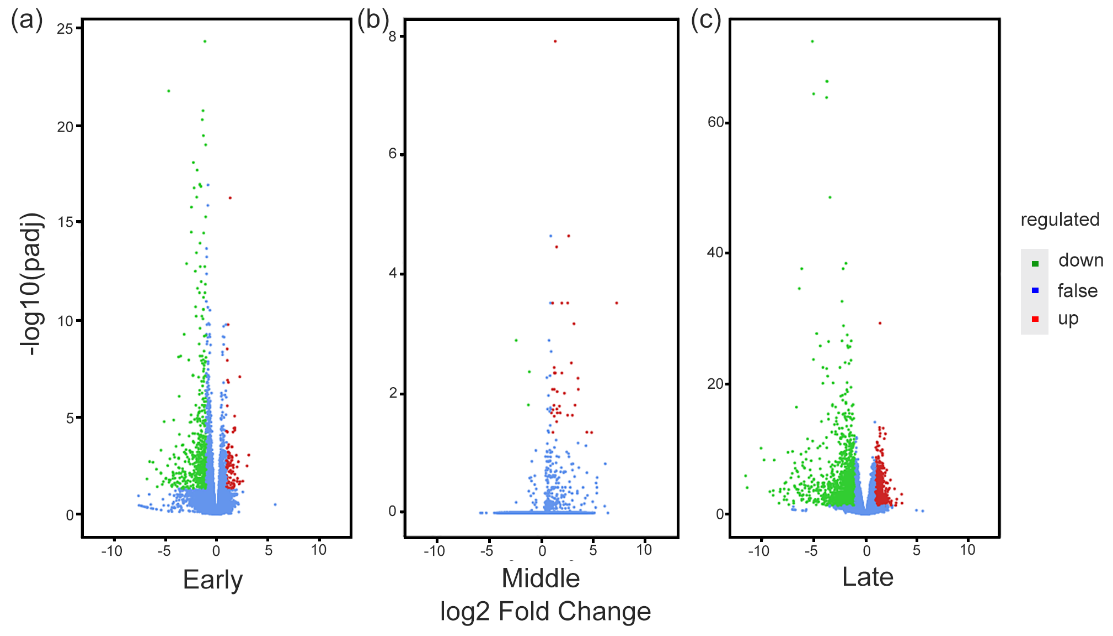


**Fig. S14** Numbers of differentially expressed genes (DEGs) detected in the three developmental stages; red indicates genes with significant up-regulation; green indicates genes with significant down-regulation; blue indicates genes with no significant change in expression.

**a b c**


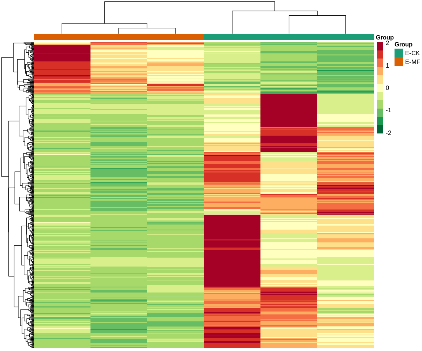

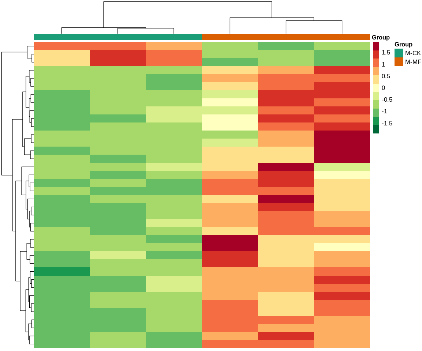

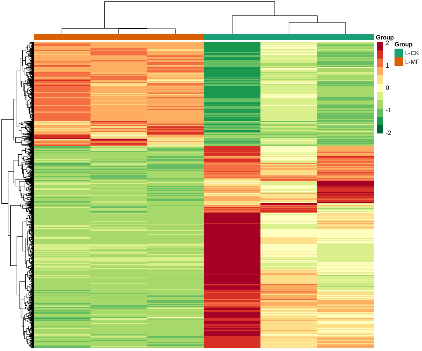
 Early Middle Late

**Fig. S15** Heat map of cluster analysis of differentially expressed genes in the three different developmental stages.


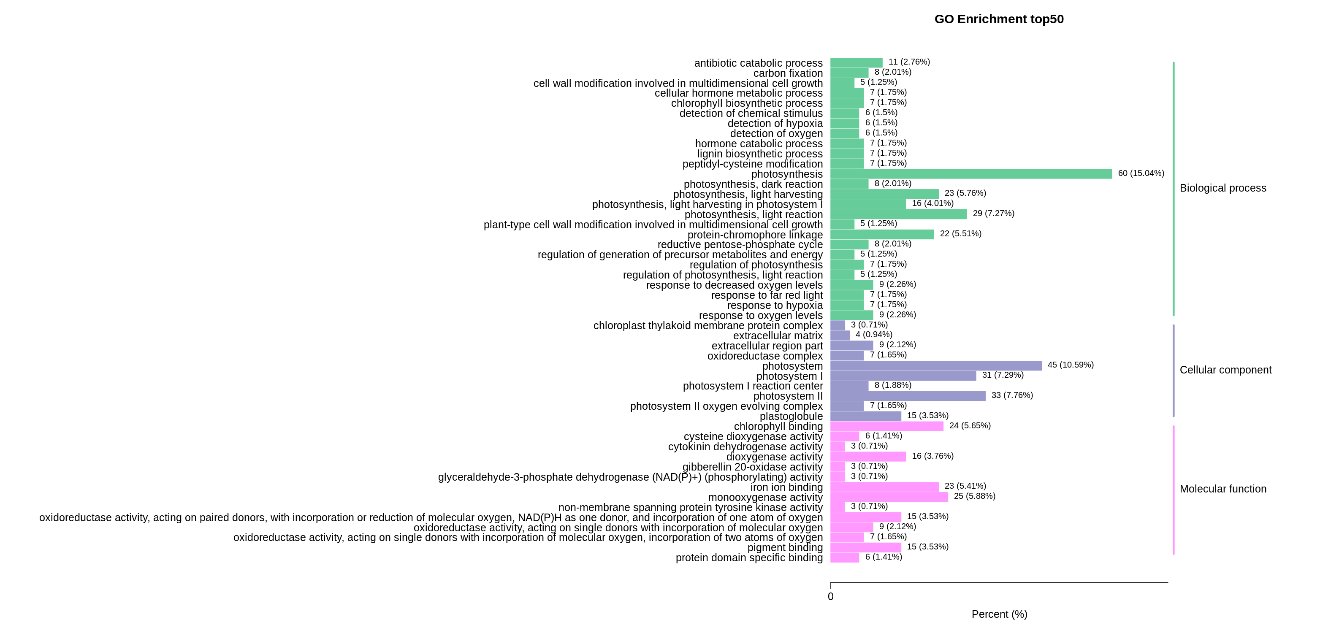


**Fig. S16** GO enrichment histogram of differentially expressed genes (early stage)


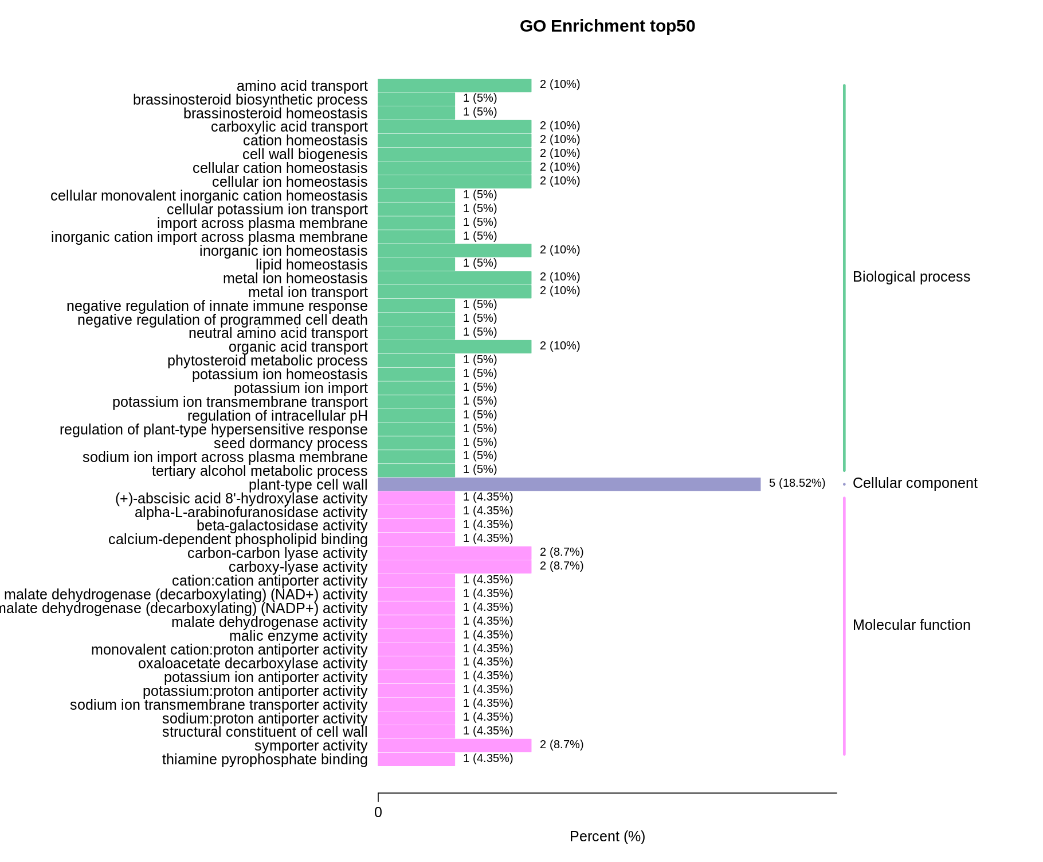


**Fig. S17** GO enrichment histogram of differentially expressed genes (middle stage)


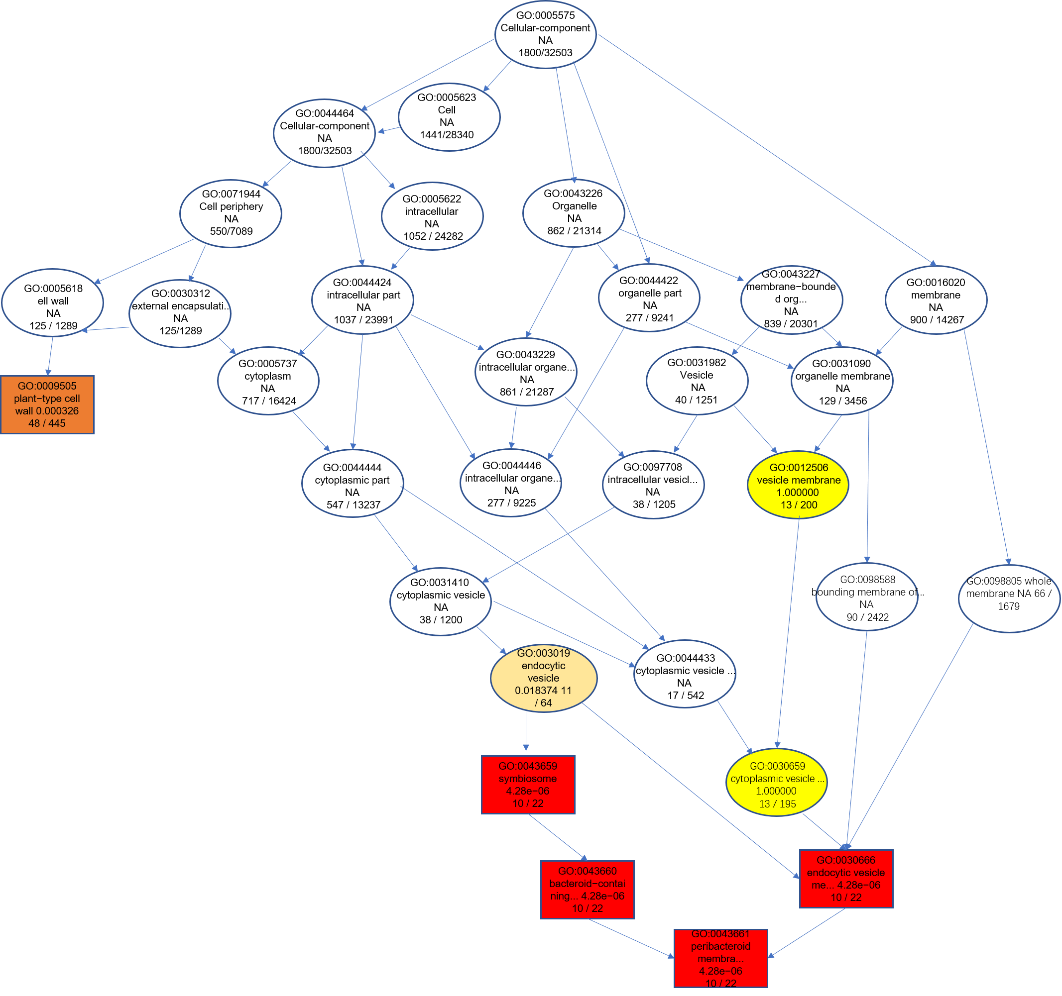


**Fig. S18** Directed acyclic graph (DAG) of enriched GO entries (late stage).

**
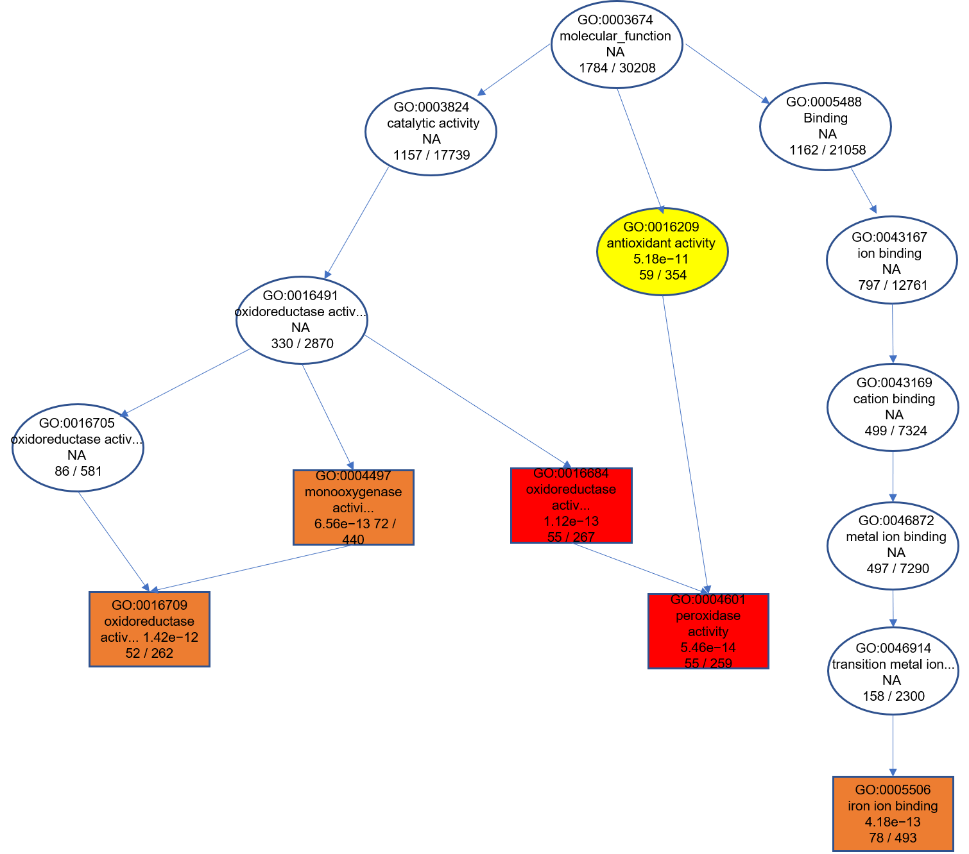
**

**Fig. S19** Directed acyclic graph (DAG) of enriched GO entries for changes in the ROS metabolism (late stage).


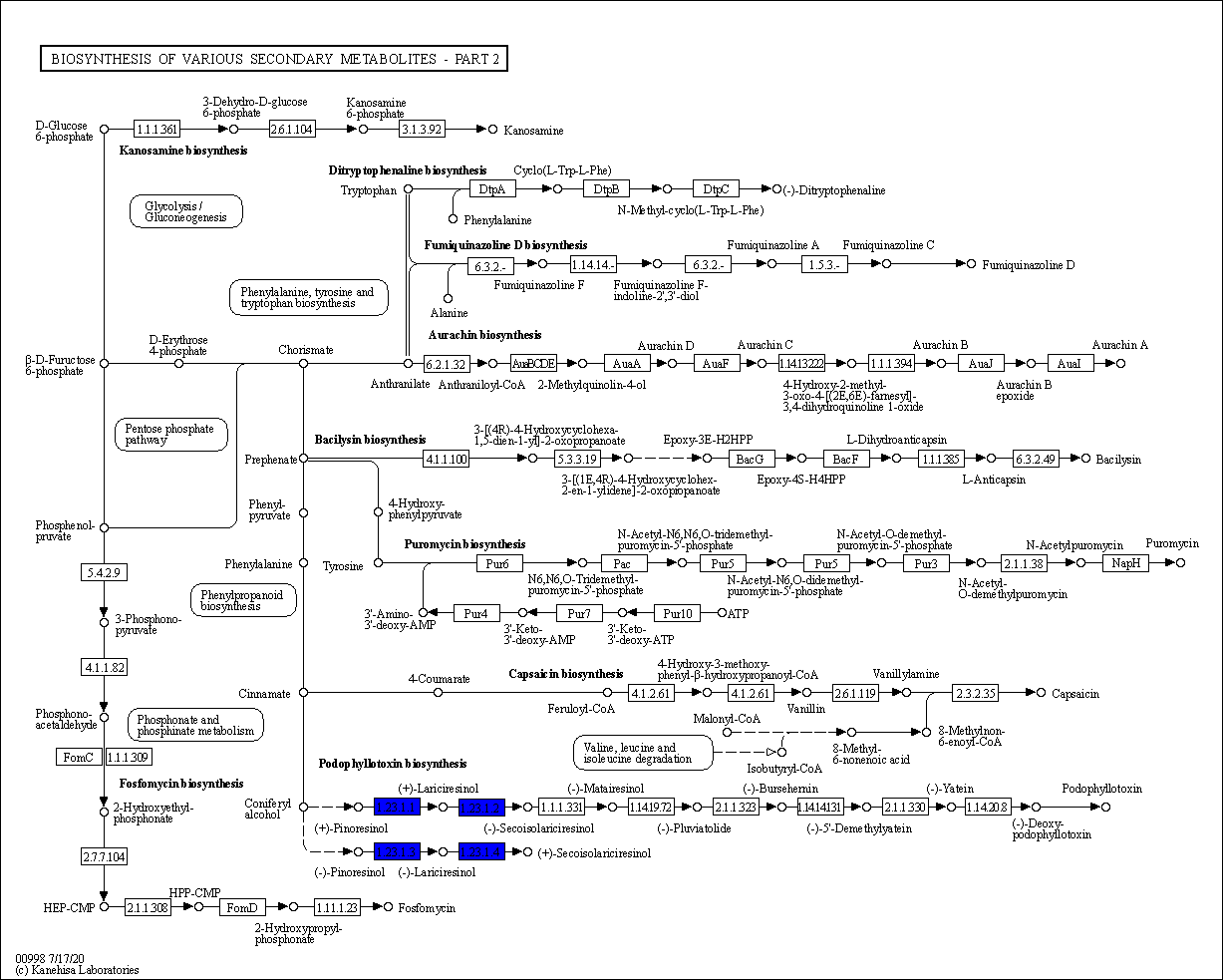


**Fig. S20** KEGG pathway of biosynthesis of various secondary metabolites - part 2 in response to MF-NPs treatment.


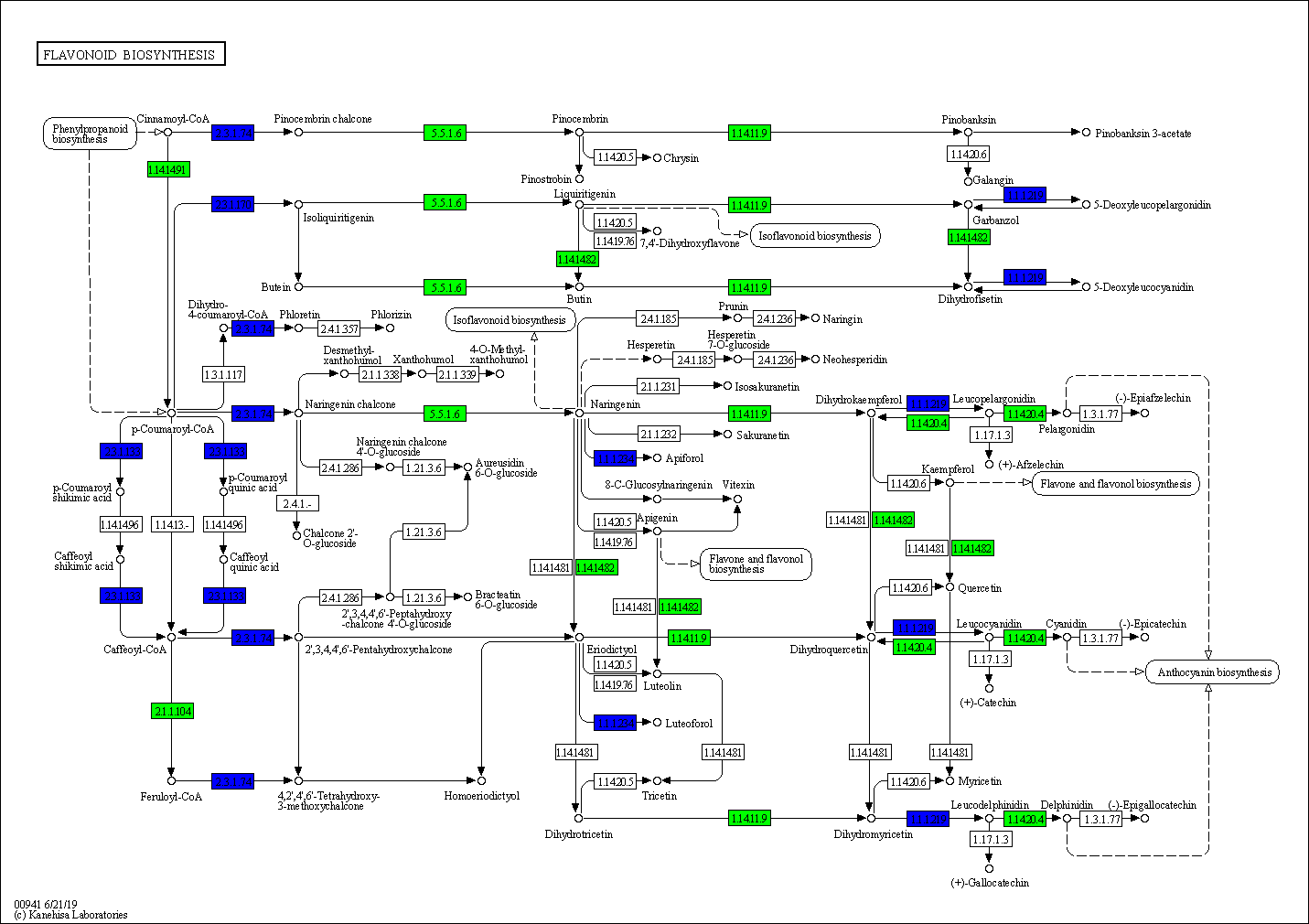


**Fig. S21** KEGG pathway of flavonoid biosynthesis in response to MF-NPs treatment.


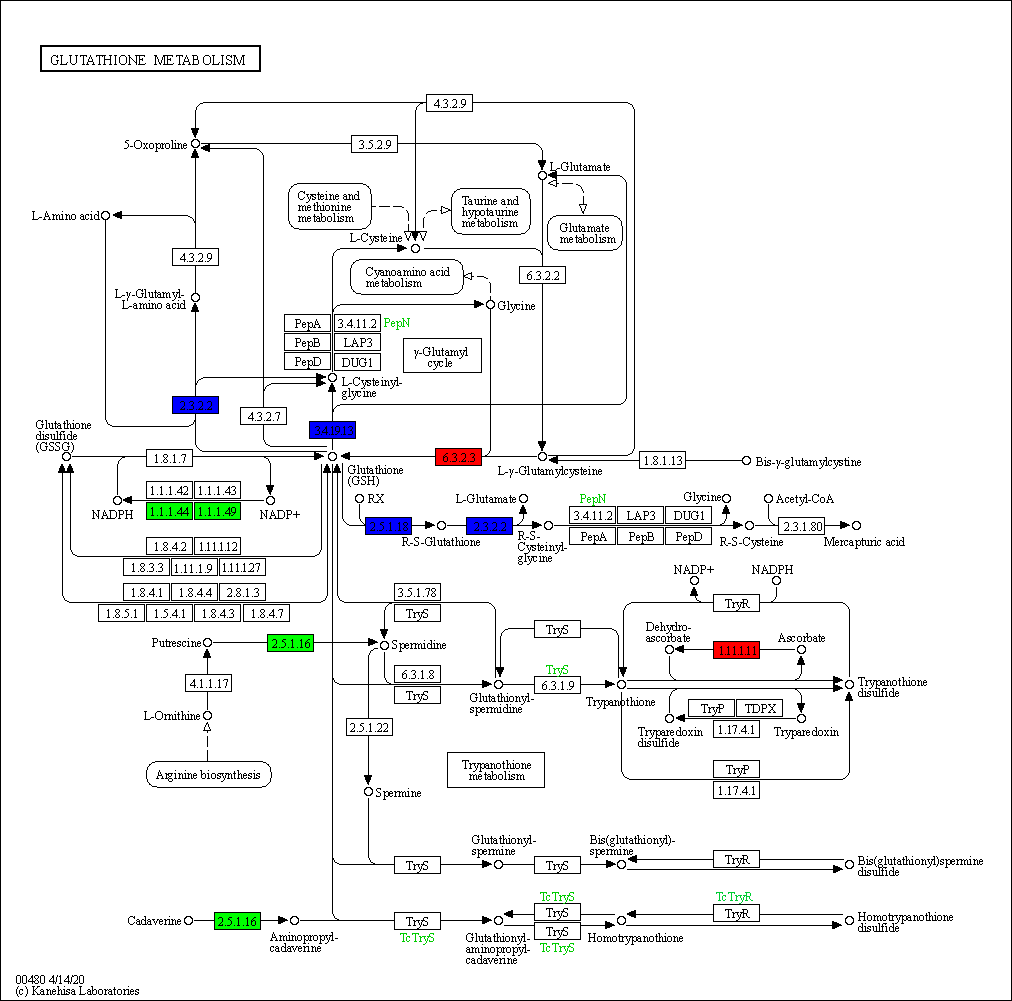


**Fig. S22** KEGG pathway of glutathione metabolism in response to MF-NPs treatment.


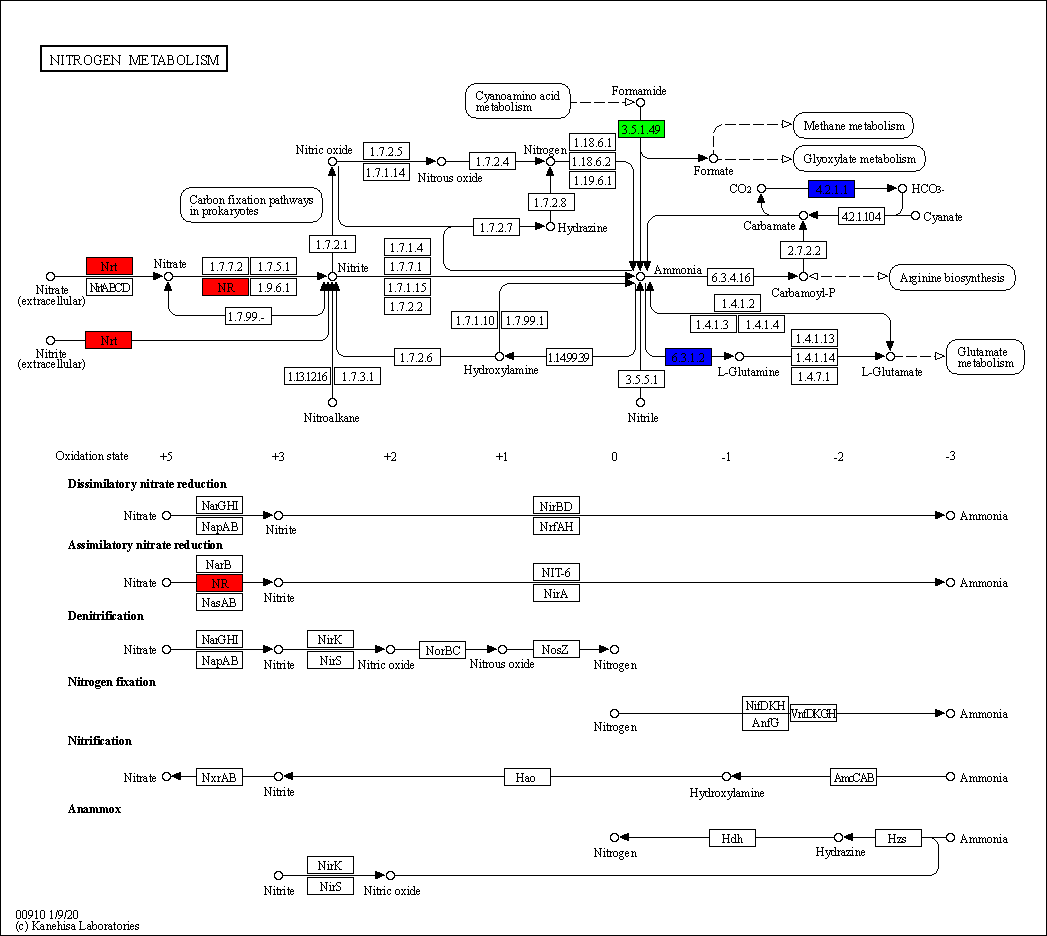


**Fig. S23** Kyoto Encyclopedia of Genes and Genomes (KEGG) pathway of nitrogen metabolism in response to MF-NPs treatment.

**Fig. S24** Standard curve of nitrogenase activity detection.

**Supplementary Table S1.** The elemental content of NPs

| **NPs** | **Element** | **Atomic percentage** |
| --- | --- | --- |
| MnO_2_ | O K | 59.88 |
|  | Mn K | 40.12 |
| Fe_2_O_3_ | O K | 47.86 |
|  | Fe K | 50.14 |
| MnFe_2_O_4_ | O K | 59.52 |
|  | Mn K | 10.84 |
|  | Fe K | 29.64 |

**Supplementary Table S2** Expression of nodulation related (Nod-R) genes in the roots in response to MF-NPs treatment.

| **Gene ID** | **Symbol** | **Up/Down Regulation** |
| --- | --- | --- |
| *ENOD55-2* | hypothetical protein GLYMA-17G073400 | up |
| *ENOD93* | early nodulin-93 | up |
| *LBA* | leghemoglobin A | up |
| *LBC1* | leghemoglobin C1 | up |
| *LBC2* | leghemoglobin C2 | up |
| *LBC3* | leghemoglobin C3 | up |
| *LOC100306363* | uncharacterized protein LOC100306363 | down |
| *LOC100527563* | uncharacterized protein LOC100527563 isoform X1 | up |
| *LOC100776166* | probable 2-isopropylmalate synthase | up |
| *LOC100778148* | probable 2-isopropylmalate synthase | up |
| *LOC100784860* | early nodulin-55-1 precursor | up |
| *LOC100791523* | nodulin-21-like | up |
| *LOC100792407* | nodulation-signaling pathway 2 protein-like | up |
| *LOC100803042* | chitin elicitor receptor kinase 1-like isoform X1 | up |
| *LOC100807538* | probable 2-isopropylmalate synthase | up |
| *LOC100816317* | nodulation-signaling pathway 2 protein-like | down |
| *LOC100817841* | early nodulin-70-like | up |
| *LOC100819730* | sucrose synthase-like | down |
| *LOC100820292* | uncharacterized protein LOC100820292 | down |
| *LOC100820501* | putative E3 ubiquitin-protein ligase LIN-1 isoform X1 | down |
| *LOC106795450* | probable 2-isopropylmalate synthase | up |
| *LOC106796780* | nodulin-16-like isoform X2 | up |
| *LOC547522* | hypothetical protein GLYMA-06G065600 | up |
| *LOC547914* | nodulin-24-like | up |
| *N-20* | hypothetical protein GLYMA-13G328800 | up |
| *N-21* | nodulin-21 | up |
| *N-22* | nodulin-22 precursor | up |
| *N-23* | nodulin-C51 precursor | up |
| *N-24* | nodulin-24 precursor | up |
| *N-26* | nodulin-26 | up |
| *N-26B* | nodulin-26B precursor | up |
| *N-36A* | early nodulin-36A | up |
| *N-44* | nodulin-44 | up |
| *N-70* | early nodulin-70 isoform X1 | up |
| *NGM-16* | nodulin-16 isoform X1 | up |
| *UR9* | uricase-2 isozyme 1 | up |

**Supplementary Table S3** Expression of flavonoid related (Fla-R) genes in the roots in response to MF-NPs treatment.

| **Gene ID** | **Symbol** | **Up/Down Regulation** |
| --- | --- | --- |
| *CHI1A* | chalcone--flavonone isomerase 1A | down |
| *CHI1B2* | chalcone--flavonone isomerase 1B-2 | down |
| *CHS3* | chalcone synthase 3 | down |
| *CHS5* | chalcone synthase 5 | down |
| *CHS6* | chalcone synthase 6 | down |
| *CHS7* | chalcone synthase 7 | down |
| *CHS8* | chalcone synthase | down |
| *CHS9* | chalcone synthase 1 | down |
| *GMACHS1* | chalcone synthase 1 | down |
| *GMCHI4A* | chalcone isomerase 4-like | down |
| *GMCHR* | NAD(P)H-dependent 6'-deoxychalcone synthase isoform X1 | down |
| *HIDH* | 2-hydroxyisoflavanone dehydratase | down |
| *LOC100194416* | vestitone reductase | down |
| *LOC100500657* | uncharacterized protein LOC100500657 | down |
| *LOC100527374* | uncharacterized protein LOC100527374 precursor | down |
| *LOC100527469* | uncharacterized protein LOC100527469 | up |
| *LOC100775264* | chalcone synthase J-like | up |
| *LOC100778253* | uncharacterized protein LOC100778253 | down |
| *LOC100779649* | chalcone synthase 5-like | down |
| *LOC100783009* | 1-aminocyclopropane-1-carboxylate oxidase 1-like | down |
| *LOC100784518* | leucoanthocyanidin dioxygenase-like | down |
| *LOC100785409* | alpha/beta-hydrolases superfamily protein | down |
| *LOC100785554* | chalcone synthase 6-like | down |
| *LOC100788048* | chalcone synthase 6-like | down |
| *LOC100789075* | chalcone synthase 3 | down |
| *LOC100790480* | flavonoid 3'-monooxygenase-like | down |
| *LOC100792581* | chalcone synthase 5-like | down |
| *LOC100793687* | uncharacterized protein LOC100793687 | down |
| *LOC100794125* | flavonol synthase/flavanone 3-hydroxylase-like | down |
| *LOC100794209* | uncharacterized protein LOC100794209 | down |
| *LOC100795616* | HXXXD-type acyl-transferase family protein | down |
| *LOC100798122* | transcription factor EGL1 isoform X1 | up |
| *LOC100800931* | NAD(P)H-dependent 6'-deoxychalcone synthase-like | down |
| *LOC100803345* | myb-related protein Myb4-like | down |
| *LOC100805464* | 1-aminocyclopropane-1-carboxylate oxidase 5-like | down |
| *LOC100805689* | cytochrome P450 711A1-like | down |
| *LOC100807814* | pathogenesis-related protein 5-like | up |
| *LOC100808297* | cytochrome P450 711A1-like | down |
| *LOC100809662* | leucoanthocyanidin dioxygenase | down |
| *LOC100815855* | ribonuclease S-2-like | down |
| *LOC100816155* | anthocyanidin 3-O-glucosyltransferase 7-like | up |
| *LOC100816546* | hypothetical protein GLYMA-09G107100, partial | down |
| *LOC100817818* | ribonuclease 1-like isoform X1 | down |
| *LOC100820161* | NAD(P)H-dependent 6'-deoxychalcone synthase-like | down |
| *LOC106794283* | chalcone synthase 3 | down |
| *LOC547660* | hypothetical protein GLYMA-18G220600 | down |
| *MYB92* | MYB transcription factor MYB92 | down |

**Supplementary Table S4** Expression of reactive oxygen species related (ROS-R) genes in the roots in response to MF-NPs treatment.

| **Gene ID** | **Symbol** | **Up/Down Regulation** |
| --- | --- | --- |
| *GMIPER1* | peroxidase, pathogen-induced precursor | down |
| *INR2* | inducible nitrate reductase [NADH] | up |
| *LOC100527587* | peroxidase 7-like protein precursor | down |
| *LOC100775837* | peroxidase 12-like | down |
| *LOC100775970* | peroxidase 3-like | down |
| *LOC100776019* | peroxidase superfamily protein | down |
| *LOC100777390* | peroxidase 12-like, partial | down |
| *LOC100777463* | peroxidase 19 | up |
| *LOC100782791* | putative L-ascorbate peroxidase 6 | up |
| *LOC100785662* | peroxidase 3-like | down |
| *LOC100786765* | peroxidase 10-like | down |
| *LOC100787073* | uncharacterized protein LOC100787073 precursor | down |
| *LOC100790188* | peroxidase 5-like | down |
| *LOC100790910* | cationic peroxidase 1-like | down |
| *LOC100791014* | peroxidase 66-like | down |
| *LOC100792351* | peroxidase 5-like | down |
| *LOC100794128* | cationic peroxidase 1-like | up |
| *LOC100796311* | peroxidase 5-like | down |
| *LOC100797524* | aspartic proteinase CDR1-like | up |
| *LOC100797875* | probable peroxidase 26 | down |
| *LOC100798064* | aspartic proteinase CDR1-like | down |
| *LOC100799162* | cationic peroxidase 1-like | down |
| *LOC100806595* | peroxidase 3-like | down |
| *LOC100799691* | peroxidase 3-like | down |
| *LOC100799894* | cationic peroxidase 1-like | down |
| *LOC100800520* | hypothetical protein GLYMA-08G097300 | up |
| *LOC100802035* | peroxidase P7-like | down |
| *LOC100803619* | peroxidase P7-like | down |
| *LOC100804126* | peroxidase 12 | down |
| *LOC100804765* | uncharacterized protein LOC100804765 precursor | down |
| *LOC100804829* | unknown | down |
| *LOC100805171* | peroxidase 10-like | up |
| *LOC100806700* | uncharacterized protein LOC100806700 precursor | down |
| *LOC100808542* | peroxidase 16-like | down |
| *LOC100810392* | peroxidase P7 | down |
| *LOC100811634* | cationic peroxidase 1-like | down |
| *LOC100811634* | cationic peroxidase 1-like | down |
| *LOC100812082* | peroxidase 55-like | down |
| *LOC100812309* | uncharacterized protein LOC100812309 precursor | down |
| *LOC100812820* | peroxidase P7-like | down |
| *LOC100812961* | peroxidase 10-like | down |
| *LOC100813471* | putative nitrate reductase 1 | down |
| *LOC100814067* | peroxidase P7 | down |
| *LOC100814964* | peroxidase N-like | down |
| *LOC100815142* | cationic peroxidase 1-like | down |
| *LOC100815350* | peroxidase 5-like | down |
| *LOC100815671* | uncharacterized protein LOC100815671 precursor | down |
| *LOC100815708* | peroxidase 10 | down |
| *LOC100817540* | uncharacterized protein LOC100817540 precursor | down |
| *LOC100817743* | L-type lectin-domain containing receptor kinase IX.1-like | down |
| *LOC100818176* | cationic peroxidase 1-like | down |
| *LOC100819100* | probable arabinosyltransferase ARAD1 | up |
| *LOC100820410* | probable peroxidase 26 | down |
| *LOC102663236* | peroxidase P7-like | down |
| *LOC102668893* | L-type lectin-domain containing receptor kinase IX.2-like isoform X1 | up |
| *LOC102670034* | peroxisomal (S)-2-hydroxy-acid oxidase GLO1-like | up |
| *LOC106796332* | peroxidase A2-like | down |
| *LOC111064645* | peroxidase family protein precursor | down |
| *LOC112997600* | hypothetical protein GLYMA-15G128800 | down |
| *LOC112997743* | uncharacterized protein LOC100814455 | down |
| *PRX4* | peroxidase precursor | down |

**References**

1. Kim J, Kim HY, Song SY, Go SH, Sohn HS, Baik S, Soh M, Kim K, Kim D, Kim HC*.* Synergistic oxygen generation and reactive oxygen species scavenging by manganese ferrite/ceria co-decorated nanoparticles for rheumatoid arthritis treatment. *ACS Nano.* 2019;13:3206-3217.

2. Kanehisa M, Goto S. KEGG: kyoto encyclopedia of genes and genomes. *Nucleic acids res.* 2000;28:27-30.

3. Bagheri H, Dyer R, Severin A, Rajan H. Comprehensive analysis of non redundant protein database. 2020.

4. Boeckmann B, Bairoch A, Apweiler R, Blatter MC, Estreicher A, Gasteiger E, Martin MJ, Michoud K, O'Donovan C, Phan I. The SWISS-PROT protein knowledgebase and its supplement TrEMBL in 2003. *Nucleic acids res.* 2003; 31:365-370.

5. Li L, Stoeckert CJ, Roos DS. OrthoMCL: identification of ortholog groups for eukaryotic genomes. *Genome res.* 2003;13:2178-2189.

6. Consortium GO.The Gene Ontology (GO) database and informatics resource. *Nucleic acids res.* 2004;32:D258-D261.

7. Finn RD, Bateman A, Clements J, Coggill P, Eberhardt RY, Eddy SR, Heger A, Hetherington K, Holm L, Mistry J. Pfam: the protein families database. *Nucleic acids res.* 2014;42:D222-D230.
